# Network-directed efficient isolation of previously uncultivated *Chloroflexi* and related bacteria in hot spring microbial mats

**DOI:** 10.1038/s41522-020-0131-4

**Published:** 2020-04-29

**Authors:** Wen-Dong Xian, Nimaichand Salam, Meng-Meng Li, En-Min Zhou, Yi-Rui Yin, Ze-Tao Liu, Yu-Zhen Ming, Xiao-Tong Zhang, Geng Wu, Lan Liu, Min Xiao, Hong-Chen Jiang, Wen-Jun Li

**Affiliations:** 1 State Key Laboratory of Biocontrol, Guangdong Provincial Key Laboratory of Plant Resources and Southern Marine Sciences and Engineering Guangdong Laboratory (Zhuhai), School of Life Sciences, School of Ecology, Sun Yat-sen University, Guangzhou, 510275 China; 2 School of Resource Environment and Earth Science, Yunnan Institute of Geography, Yunnan University, Kunming, 650091 China; 3 State Key Laboratory of Biogeology and Environmental Geology, China University of Geosciences, Wuhan, 430074 China

**Keywords:** Environmental microbiology, Bacteriology

## Abstract

The perplexity of the complex multispecies community interactions is one of the many reasons why majority of the microorganisms are still uncultivated. We analyzed the entire co-occurrence networks between the OTUs of Tibet and Yunnan hot spring samples, and found that less abundant OTUs such as genus *Tepidimonas* (relative abundant <1%) had high-degree centricity (key nodes), while dominant OTUs particularly genus *Chloroflexus* (relative abundant, 13.9%) formed the peripheral vertexes. A preliminary growth-promotion assay determined that *Tepidimonas* sp. strain SYSU G00190W enhanced the growth of *Chloroflexus* sp. SYSU G00190R. Exploiting this result, an ameliorated isolation medium containing 10% spent-culture supernatant of *Tepidimonas* sp. strain SYSU G00190W was prepared for targeted isolation of *Chloroflexi* in the Tibet and Yunnan hot spring samples. 16S rRNA gene fingerprinting characterized majority of the colonies isolated from these media as previously uncultivated *Chloroflexi*, of which 36 are potential novel species (16S rRNA sequence identity <98.5%). Metabolomes studies indicated that the spent-culture supernatant comprises several low-molecular-weight organic substrates that can be utilized as potential nutrients for the growth of these bacteria. These findings suggested that limited knowledge on the interaction of microbes provide threshold to traditional isolation method.

## Introduction

Earth is by large a microbial world. About 10^6^ prokaryotic species were estimated to exist on Earth^1^, of which ~12,000 (~1% of the total) species have so far been cultivated and validly described^2^. The remaining ~99% either fail to grow on the synthetic growth media used in the laboratory or require special or unidentified culture techniques^3^. We do, however, understand that these uncultured microbes play unprecedented roles in carbon and nitrogen cycling, novel natural products chemistry, and in maintaining the balance of the environment.

Factors influencing the cultivability of these uncultivated microorganisms have been extensively studied. The inclusion of next-generation sequencing technology improvised our understanding on the community-wide microbial diversity. Despite the added advantages, it is still a challenge to cultivate the majority of the microbial population. In some cases, the synchrony of the detected species between culture-independent and culture-dependent techniques were as low as 15%^4^.

Majority of the estimated microorganisms in natural environments exist in the form of biofilms^5^, and in totality, they account for ~80% of available prokaryotic cells^6^. Microbial biofilm is widely distributed in environments^7^, including terrestrial habitats^8,9^, water^10,11^, and human microcosms^12,13^. The terrestrial hot spring is a unique ecosystem for “extremophiles”, and has long been considered as a model habitat for exploring principles of microbial ecology^14–17^. Hot spring microbial mats (HSMMs) represent the most complex community, in which bacterial cells stick to each other and the adherent cells embedded within the extracellular matrix. HSMMs are usually close self-sustaining small ecosystems, with total inclusion of basic biogeochemical cycles and food chains^18^. The structure of these communities was well described in previous studies^18–20^. The dominant members of these HSMMs are usually the filamentous anoxygenic phototrophs (FAPs), comprising of *Chloroflexus* spp. and *Roseiflexus* spp. of the phylum *Chloroflexi*. Primary productions in this habitat are generally driven by FAPs and *Cyanobacteria*^18^, but in certain cases, it is only the FAPs that reside therein^19^. Further, they are the renewable biomass resource for the production of industrial chemicals; for instance, 3-hydroxypropionate, an intermediate of the hydroxypropionate/hydroxybutyrate cycle in FAPs, is an important precursor for the synthesis of bioplastics^21^. Majority of the FAPs and related bacteria have still not been cultivated, and the lack of culture is the main hindrance in understanding the community structure and mechanism of the biogeochemical cycles in this environment.

Co-occurrence network is routinely used to analyze the potential interactions among community members, to seek microbial social modules and to estimate the importance of a particular operational taxonomic unit (OTU) in a community^22,23^. We hypothesize that “key node” bacteria with high-degree centricity in a microbial community network play the role of organizing and regulating the community by facilitating the neighboring members, using any of the following mechanisms: (1) decompose organic matters (including apoptotic bacterial cell) produced by autotrophic cells to provide essential nutrients for other members; (2) produce acidic or alkalescent metabolites during the cell growth to buffer the pH value of the environment^24^; (3) regulate the environmental adaptability of other bacteria by providing specific nutrients, such as some growth factor^25^; (4) eliminate toxic metabolites like H_2_O_2_^26^ to facilitate cells from inhibitory stress; (5) supply quorum-sensing signal molecules to induce colony formation^27^. In this study, we processed a “key node” growth-promoting species to target and isolate previously uncultivated strains of the phylum *Chloroflexi* within HSMMs. Samples investigated in this study include a large number of hot springs located in Southwestern China.

## Results

### Environmental parameters of the sampled hot springs

**Fig. 1:**
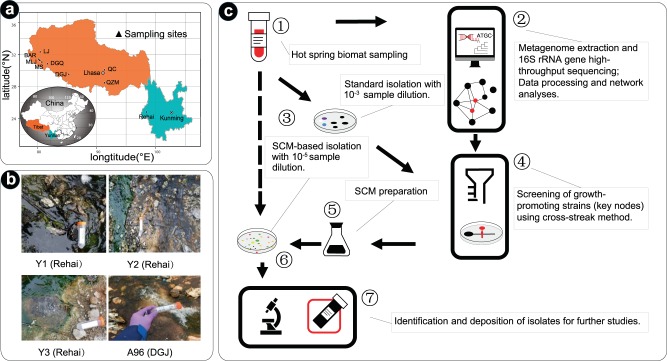
Overview of the sampling sites and the workflow for the network-directed isolation procedure from HSMMs. **a** Location of the sampling sites; **b** representative samples (Y1, Y2, and Y3 from Yunnan, and A96 from Tibet hot springs) that are used for isolation; **c** workflow use to predict key-node taxa and screening for growth-promoting strains. SCM plate was effective in the isolation of targeted microorganisms from the HSMMs.

The physicochemical parameters of the hot springs selected for this study are listed in Supplementary Table 1. The temperature and pH recorded in these hot springs were in the range of 45.0–71.7 °C and 6.5–8.0, respectively. The hot springs of Qucian (QC), Quchomo village (QZM), Kunggyu geothermal area (DGQ), Moincer (MS), Molo’gyam (MLJ), and Langju geothermal area (LJ) presented a circumneutral pH (6.5–7.0), while Bar hot spring (BAR) and Daggyai geothermal area (DGJ) were nearly neutral to slightly alkaline (pH 6.5–8.0). The three sampling sites in Rehai National Park (Y1, Y2, and Y3) are newly formed HSMMs but had been meddled with human activities, possibly during the construction and subsequent renovation of the scenic spot. The sample A96 from DGJ, Tibet (52 °C, pH 8.0; Fig. 1) is a well-developed HSMM measuring 2–3 cm in thickness, and no sign of human activity was detected during our sampling.

### Community structure and co-occurrence network analysis

**Fig. 2:**
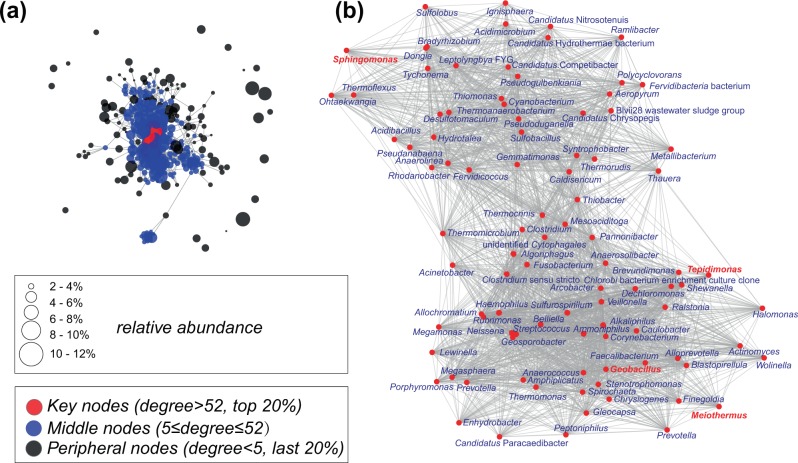
Microbial co-occurrence networks analysis. **a** Co-occurrence networks based on high-throughput sequencing of communities in 26 hot spring microbial mat communities. The relative abundance and degree centrality characteristics were mapped to each vertex; key nodes are colored with red, and middle nodes with blue. **b** All 90 key nodes are separated from the whole network. Among them, isolates belonging to four genera (red font) were previously cultivated by our laboratory, and were treated as potential growth-promoting strains in this study.

A total of 2,173,872 quality barcode amplicons reads were obtained from 26 HSMM samples with an average of 83,610 sequence reads per sample (Supplementary Table 2). These sequence reads were clustered into 1674 OTUs at the specific level. Following filtering of the OTUs related to uncultured bacteria, 456 OTUs were annotated at the generic level. Ninety of these OTUs were determined as key nodes in the co-occurrence network analysis (Fig. 2). The mean relative abundance of the majority of these genera was <1% with the exception of OTUs related to *Meiothermus* (Fig. 2b). The peripheral nodes in the network comprised 253 OTUs, with the genera *Chloroflexus* (13.9%), *Thermus* (11.05%), and *Roseiflexus* (8.44%) accounting for 64.8% of the total peripheral nodes (Fig. 2b).

### Key-node species and spent-culture medium (SCM) preparation

**Fig. 3:**
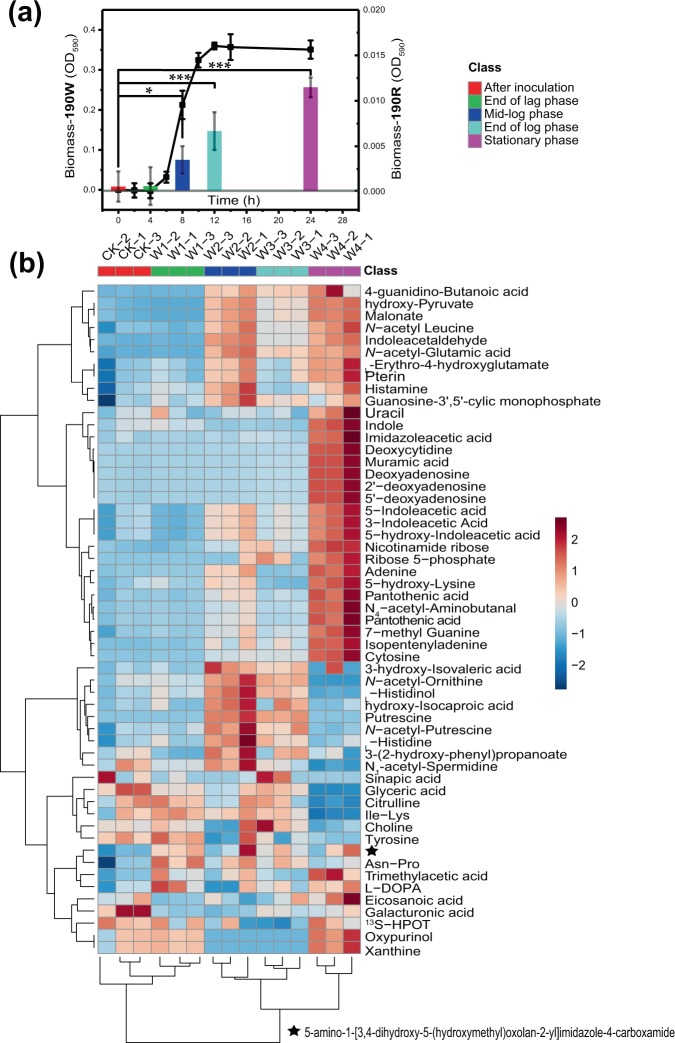
Metabolome analysis. **a** Growth curve of *Tepidimonas* sp. strain **190W** (line plot), and growth-promoting effects by 10% spent-culture supernatants of *Tepidimonas* sp. **190W** (histograms indicating increase in biomass of *Chloroflexus* sp. strain **190R** at different time intervals). Error bars represent standard errors. *p < 0.05 and ***p < 0.001. **b** Heatmaps indicating the concentration of different extracellular metabolites released by *Tepidimonas* sp. strain **190W** at different growth phases. Each column represents one biological replicate, and each row a targeted metabolite detected in this study. The hierarchical clustering on the left represents the closely related metabolites accumulation with similar growth trend, and those on the bottom the closely related growth stage with similar metabolites accumulation. The color scale represents the scaled abundance of each metabolite, denoted as d 2 (squared Euclidean distance), with red indicating high abundance and blue indicating low abundance. The compounds represented in the heatmap are listed in Supplementary Table 5. All samples were from three biological replicates.

Among the OTUs identified as key nodes in the entire network, members of the genera *Tepidimonas*, *Geobacillus*, *Meiothermus*, and *Sphingomonas* (Supplementary Fig. 1) have been previously isolated by our laboratory from hot spring samples using traditional agar plate method. For preparation of SCM, a preliminary growth-promotion assay of these key-node species from our culture collection was performed. These species had different growth physiology, e.g., *Sphingomonas* sp. SYSU G00007 grew at 45 °C but not at 55 °C, while *Meiothermus* sp. SYSU GA4201 grew at both the temperatures. For the growth-promotion assay, a slow-growing *Chloroflexus* sp. strain SYSU G00190R (also isolated during another earlier study; hereafter referred to as **190R**) was selected as the indicator organism. The indicator *Chloroflexus* sp. strain **190R** showed closed similarity (99.2% 16S rRNA gene sequence identity) to peripheral node bacterium *Chloroflexus islandicus* isl-2 (LWQS01000112). The *Chloroflexus* sp. strain **190R** grew at both 45 °C and 55 °C after 1-week incubation under dark conditions. In the growth promotion assay using a cross-inoculation method, the growth of *Chloroflexus* sp. strain **190R** was promoted when inoculated along with *Tepidimonas* sp. SYSU G00190W (referred hereafter as **190W**; Supplementary Fig. 1). The *Chloroflexus* sp. strain **190R** showed good growth on R2A agar supplemented with 10% and 20% spent-culture supernatants of *Tepidimonas* sp. strain **190W**, moderate growth when supplemented with 1% and 5% spent-culture supernatant, but not on the plates induced with cell-free extract (Supplementary Fig. 2). Of five harvesting time periods tested, supernatants collected at 8, 12, and 24 h produce significant growth enhancement (*p* < 0.05) to *Chloroflexus* sp. strain **190R** (Fig. 3a). Among the three time intervals, the growth enhancement by 12 and 24 h supernatants on *Chloroflexus* sp. strain **190R** was significant at *p* < 0.001 as well. Based on the findings of the above assays, the 24 h spend culture supernatants of *Tepidimonas* sp. strain **190W** was selected for preparation of spent-culture medium (SCM) for use in the isolation and culturing of the slow or previously uncultivated bacteria particularly related to the phylum *Chloroflexi* from the HSMM samples.

### Standard media- vs SCM-based isolation

**Fig. 4:**
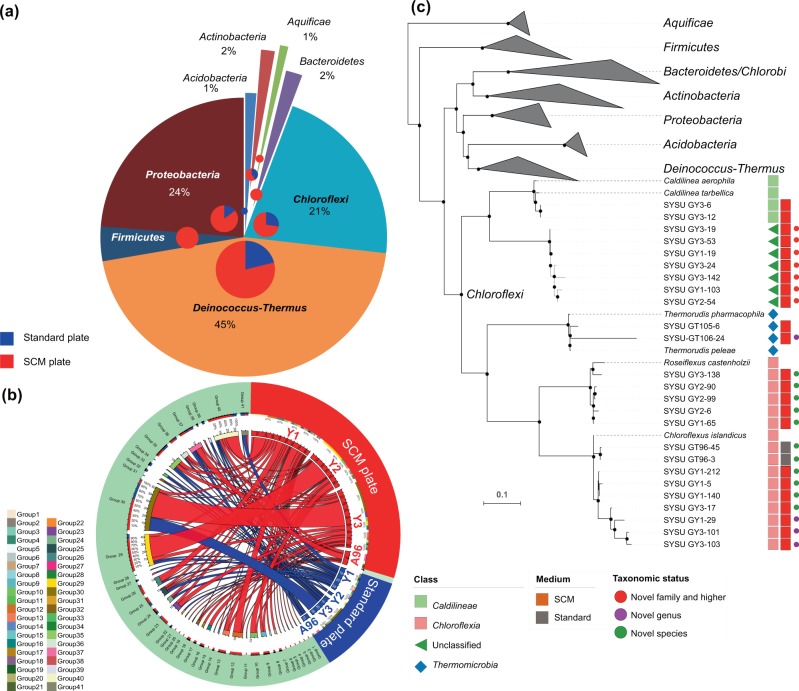
Isolation efficacy of previously uncultured *Chloroflexi* and related bacteria on spent-culture media. **a** Distribution of isolates determined from the samples; smaller pie-chart represents the isolation media. **b** Circos plot showing the alignment between samples (right), and strains isolated (left). The sample contigs are depicted in red (experimental group) and blue (control) color scheme, and contigs depicting the taxonomic group in rainbow colors. **c** Maximum-likelihood tree indicating the phylogenetic relationship of representative strains isolated in this study. Symbols following the strain names represent the relative affiliation at class level, blocks the isolating medium, and color circles the putative taxonomic ranks for the representative taxa. Bar, 0.1 substitutions per nucleotide site.

Applying the traditional isolation method on the standard isolation media (Supplementary Table 3) and on SCM, a total of 319 isolates distributed in eight phyla (4 *Acidobacteria*, 5 *Actinobacteria*, 3 *Aquificae*, 6 *Bacteroidetes*, 69 *Chloroflexi*, 142 *Deinococcus-Thermus*, 13 *Firmicutes*, and 77 *Proteobacteria*) (Fig. 4c; Supplementary Fig. 3; Supplementary Table 4) were isolated from the HSMM samples. Seventy-five percent (239 strains) of these strains were isolated using the SCM plates. Members of the phylum *Chloroflexi* were more frequently isolated on the SCM plates (57) in comparison with the standard isolation media. Further, the *Roseiflexus* spp. and *Chloroflexus* (Group 14 and 15; Fig. 4b) were isolated only on the SCM plates. The Group 40 (closely related to *Chloroflexus islandicus*, Fig. 4b) was, however, isolated on both the standard and SCM plates, but the growth was most prominent on the SCM plates (Supplementary Fig. 4). When the isolates with low 16S rRNA gene identities (<90%) were cloned and the almost full-length sequences determined, four isolates presented distinct lineages in the pre-aligned ARB tree (SILVA LTPs132_SSU database) (Supplementary Fig. 5). Two of these isolates belong to the phylum *Chloroflexi*, and the other two of the phyla *Bacteroidetes* and *Chlorobi*.

Efficacy of the SCM-based isolation was validated by subculturing all the 113 potential novel bacterial species isolated in the study onto R2A agar, T5 agar, CC agar, and *Thermus* 162 agar media (Supplementary Table 3), all of which are effectively used for growing thermophiles. All the strains effectively grew on the SCM plates, while limited strains could grow on *Thermus* 162 agar (5 strains), R2A agar (8 strains), and none on either T5 agar or CC agar.

### Comparative metabolomics of spent cultures

As the growth promotion on *Chloroflexus* spp. was observed with SCM, it was hypothesized that *Tepidimonas* sp. may have a strong ability to synthesize and excrete compounds. To test the hypothesis, comparative analyses were conducted between *Tepidimonas* and *Chloroflexus* genomes. The genomes of *Tepidimonas* have significantly higher (*p* < 0.001) genes related to transport and binding proteins as compared with *Chloroflexus* (Supplementary Fig. 6), but the metabolites excreted were still beyond our speculations. The full range of low-molecular-weight organic substances (LMWOS) of *Tepidimonas* sp. strain **190W** was determined by analyzing the non-targeted extracellular metabolomes collected at 0, 4, 8, 12, and 48 h (see “Methods”). Of 412 compounds detected in the extracts, 83 were significantly accumulated (*p* < 0.05) (Fig. 3b; Supplementary Table 5). Amino acids and analogues were accumulated mostly at the end of the lag growth phase (4 h). In the exponential phase (8 h), concentration of these compounds decrease, and synchronically the compounds isopentenyladenine, cytosine, pyrrole-2-carboxylic acid, and hydroxypyruvate started accumulating. At the end of the exponential growth phase (12 h), components like adenine decrease to a very low level (from fivefolds to onefold). After another 12 h in stationary phase (24 h), a large number of small molecular compounds started accumulating. These include compounds of the following categories: nucleosides and analogues, indoles and derivatives, organic acids and derivatives, organic oxygen compounds, organoheterocyclic compounds, pteridines and derivatives, purines and purine derivatives, and quinolines and derivatives. Accumulation of the nucleosides and analogues (2–78.9-folds increase) were most prominent among all the categories of the LMWOS, particularly the deoxycytidine (a 78.9-folds increase). The other compounds in this category include 2′-deoxyadenosine, 5′-deoxyadenosine, 3′-deoxyadenosine, cytosine, thymidine, adenine, cytarabine, and cytidine. In addition, indoles and derivatives (well-known growth regulators), including indole acetaldehyde, indole, melatonin, uracil, 3-indoleacetic acid, and 5-hydroxyindoleacetaldehyde, were accumulated during this phase. The proportion of indole, melatonin, and uracil was remarkably accumulated (3.3–13.4-folds; *p* < 0.001) in the stationary phase when compared with their concentrations at the end of the exponential growth phase (12 h).

### Growth-promotion assay

Fifteen commercially available metabolites (Supplementary Table 6) that were found accumulated in the **190W** supernatant were procured and tested for growth promotion using nine representative *Chloroflexi* and *Bacteriodetes* strains isolated in this study (Supplementary Fig. 7). Among these compounds, pantothenic acid (VB5) and 3-indoleacetic acid (IAA) facilitate growth promotion of all the tested strains (Supplementary Fig. 7). Imidazole acetic acid and deoxycytidine showed enhance growth to *Chloroflexus* strains, while putrescine improved the growth of *Roseoflexus* strains. Further, uracil, cytosine, and adenine also facilitate the growth of other *Chloroflexi*, but the enhanced growth OD of the representative *Bacteroidetes* isolate was observed only with VB5.

## Discussion

Although sufficient efforts have been made in culturomics, the “Great Plate Count Anomaly”^28^ is still a puzzle, and hitherto uncultured microorganisms are vividly imaged as microbial “dark matters”^3^. For a long period, the attributes of the microbial community interactions were not fully considered. Microorganisms thrive in complex multispecies communities rather than as separate entities under in situ environments, and complex interactions take place frequently among inhabitants. Cell to cell distances in HSMMs is short enough (~100 μm) for diffusible metabolites to reach the neighboring cells within the microscale cell aggregates^29^. Bacterial interactions in the community can determine the fate of bacterial populations^24^, which is the most complex environmental factor because it is elusive, and varies as members interact with different community members, besides altering the chemicals response during the interactions. Microbial mats in the effluent channels of the alkaline siliceous hot springs are among the most intensively studied natural microbial communities and serve as model systems for exploring general ecological principles in microbial ecology.

Construction of network with OTUs at the generic level avoids the effects incurring from important but uncultured OTUs. Filtering of these unclassified OTUs also resulted in the filtering of some key node bacteria. This process however guaranteed that all the top identified OTUs are easily cultivated microbes. Another advantage of selecting the top centric key nodes is that isolates such as members of the genus *Anaerosolibacter* that are strictly anaerobic were filtered and that all the isolation and culturing employed in this study was conducted in aerobic conditions.

As opposed to our original proposition of using the network modularity to predict nodes directly connected with the genus *Chloroflexus* into a module subnetwork with closely associated members, OTUs of the genus *Chloroflexus* were found mostly on the peripheral node in the entire network, and were not admitted into any module. This finding suggested that the community attributes of *Chloroflexus* may not be obvious in the network, and needs facilitation from other members. To further explore the characters of these vertexes, the abundance of OTUs was mapped to each node. The abundant members particularly genera *Chloroflexus* and *Roseiflexus* were distributed as peripheral vertexes, while the key nodes were largely members of rare bacterial taxa particularly of genus *Tepidimonas* (relative abundance, 0.014%). We concluded that the abundant members attributing significant ecological function may not be robust in community regulation, such as transfer of small molecular metabolites that are necessary for cell–cell interaction. On the contrary, the microbial community within HSMMs was sustained by the rare members with high centrality by facilitating the basic metabolisms of abundant members. A similar observation was made in Lake Ecosystem as well^30^. We, therefore, select the key nodes with top 20% degree centrality i.e., have most links with other members in the network, as potential growth promoter.

Over the past several years, several culturomics strategies have been designed to increase the efficacy of isolation of previously uncultured members of microbiota in different environments. Some focused on improving the isolating media, by making the components similar to the sample condition^31–34^, while others physically separated cells to avoid microbial competition and allowed the growth of slow-growing bacteria^35,36^. Few studies inferred the biological features from genome data^37–39^ to target a particular bacterium, while some considered specific factors, such as catalase or pyruvate to eliminate growth stress^40,41^. Few groups further assessed by modifying the methods of media preparation to effective culture different microorganisms^42–45^, while others emphasized on the role of growth factors released by partner bacterium for cultivation of previously uncultured spp^46^. However, discoveries of novel strains through these methods are getting limited, which greatly hinder our understanding of microorganisms and their environmental processes.

As there is no report of using either network-directed isolation techniques, or successful isolation if attempted earlier, we focus this study on the community attributes of microorganisms. Microorganisms do not exist as a single entity in the in situ environment, but tend to live in communities consisting of myriad diverse and interacting species. For individuals therein, interspecific interactions like cross-feeding occurred frequently; as a result, the surrounding microorganisms represent the most complex and critical environmental factor. Microorganisms have complex interactions, such as symbiosis, cooperation, and competition. In the process of long-term cooperative coevolution, some microbes result in dramatic differentiations, including loss of function as part of their need to grow and adopt basic biological metabolism ability. Approximately 98% of the environmental microbes are auxotroph^47^, and promote the sociality between microbes through collaboration. This network is the foundation of the microbial diversity and community stability.

In the co-occurrence network map (Fig. 2), a total of 90 key nodes were confirmed, of which four nodes related to genera *Geobacillus*, *Meiothermus*, *Sphingomonas*, and *Tepidimonas* were mapped to previously isolated strains in our collection. Members of these genera have been previously reported as prototrophic microbes producing potential growth-promoting factors^48–50^, Two of the genera *Tepidimonas* and *Geobacillus* showed strong growth-promotion activity on *Chloroflexus* sp. strain **190R** (Supplementary Fig. 1). On the other hand, *Sphingomonas* sp. SYSU G00007 and *Meiothermus* sp. SYSU GA4201 showed only weak growth-promotion activities on *Chloroflexus* sp. strain **190R**. One probable reason is that *Sphingomonas* sp. SYSU G00007 showed growth only at 45 °C and not at 55 °C, the optimal growth temperature for *Chloroflexus* sp. strain **190R**. As for the *Meiothermus* sp. SYSU GA4201, despite having good growth at 55 °C, it did not show obvious activity at the edge of ecological amplitude which may be related to the limited predictability of network employed in this study.

Between the *Tepidimonas* and *Geobacillus* isolates, the *Tepidimonas* strain **190W** secretes more growth-promoting biomass that spread over a wide spectrum of metabolites, and was selected as targeted strain for SCM preparation. The devised medium SCM was highly successful in cultivation of many previously uncultured bacteria, including members of the targeted phylum *Chloroflexi*. The presentation of two further distinct lineages of the phyla *Bacteroidetes* SYSU GT96-39-2 and *Chlorobi* SYSU GT96-40-1, besides the two distinct lineages of the targeted *Chloroflexi*, suggested that effects of the spent-culture supernatants in SCM were not restricted only to the targeted organisms but also to other hard-growing microbes.

An important aspect of this study was that supernatant, rather than the intracellular extracts, was more important in promoting the growth of indicator organisms. The growth of *Chloroflexus* sp. strain **190R** was strongly facilitated by spent-culture supernatant of *Tepidimonas* strain **190W**, but not by cell-free extracts (Supplementary Fig. 4). This finding suggested that the effective constituents are not the remnants of apoptotic cells, but the active metabolites secreted by the effector organisms. This conclusion was further confirmed by the comparative analyses between the genomes of *Chloroflexus* spp. and *Tepidimonas* spp., including characteristics involved in intracellular transport and cellular secretion. Functions of each protein-coding gene were analyzed based on the TIGR assignments. The *Tepidimonas* genomes have significant higher (*p* < 0.001) genes coding for transport and binding proteins in comparison with *Chloroflexus* (Supplementary Fig. 6). We assumed that these gene products of the *Tepidimonas* spp. might facilitate the secretion of diverse secondary metabolites into the surrounding extracellular space, which is then taken up by the other targeted microbes as nutrient resources or growth factors. This assumption is consistent with the diverse metabolites detected in the supernatant from *Tepidimonas* sp. strain **190W**.

Metabolome analysis of the supernatant in stationary growth phase detected the accumulation of large numbers of metabolites. Few of the LMWOS were frequently reported as necessary nutrients for the growth of bacteria^34^. In our experimental verification, the increase in growth OD of all representative strains by supplementation of VB5 and IAA proved that production of these growth-promoting factors in the spent culture have positive impact on bacterial growth, possibly facilitating the isolation of previously uncultivated bacteria^51–54^. All the tested compounds showed different degree of activities against selected bacteria (Supplementary Fig. 7). As for instance, cytosine greatly improved the growth of strain *Chloroflexus* sp. GY3-101, but not strain *Chloroflexus* sp.SYSU GY1-5 (Supplementary Fig. 7). Uracil, cytosine, and adenine (compounds necessary for cell division during cell proliferation) facilitated the growth of most of the tested strains. We could not, however, predict any correlation between the concentration of the metabolites and its growth enhancing effect. The two most accumulated metabolites deoxycytidine (78.9-folds increase) and isopentenyladenine (44.8×) were reported as effective growth promoters^55–57^, but none were found to have enhance effect in our study. Besides, this study was limited to screening of their individual effectiveness, and as a result correlation effect of different combination of these compounds and possible intra- and interspecies chemical signaling between different microorganisms could not predicted from this limited study. At the most, we could say that the metabolites secreted by *Tepidimonas* sp. strain **190W** contained a large variety of bacterial growth-promoting or regulating compounds, which enhances the culturing of many previously uncultivated bacteria.

Our work further provides strong experimental evidence that key-node bacteria is critical for rapid and easy cultivation of hard and slow-growing strains in the laboratory condition. By providing evidence for the relationship between *Tepidimonas* and *Chloroflexus* isolates, our study further advances the understanding of the critical growth-promoting roles played by *Tepidimonas* in the isolation of *Chloroflexi* from HSMMs. In summary, network-directed isolation using SCM is effective for obtaining previously uncultivated bacteria from HSMMs.

## Methods

### Field measurements and sample collection

A total of 26 HSMMs samples were collected from Tibet (August 2015, August 2016, August 2018) and Yunnan (December 2017), southwest China. These samples were distributed into nine sampling areas: QZM, QC, DGJ, DGQ, MS, MLJ, BAR, and LJ in Tibet and Rehai (Y1, Y2, and Y3) in Yunnan (Fig. 1a; Supplementary Table 1). Most of the sampling sites are small pools with an outflow path, along which good biofilms or mats are developed. Both temperature and pH of the sampling sites were measured in situ. After the field environmental parameters were measured, the microbial mat samples (including few sediments) were collected into 50-mL sterile sample tubes, and placed either on dry ice (used for environmental DNA extraction) or kept at room temperature (for bacteria isolation). The sample marked A96 from DGJ include both from the center and bottom of the middle outflow of the biggest geyser (Fig. 1b). The Rehai sample Y1 was a dark green steam mat located 1 mile away from the hot spring source, Y2 a brownish-red mat stick on the bottom of the steam, and Y3 a yellow biofilm in the outlet and green cyanobacteria-like mats of the surrounding. For this study, all samples were used for high-throughput sequencing, network analysis and isolation of potential growth-promoting strains, while samples Y1–Y3 from Yunnan and A96 from Tibet were used for bacterial isolation.

### High-throughput sequencing and co-occurrence network analysis

The total DNA was extracted from 0.5 g samples using the Fast DNA SPIN Kit for Soil according to the manufacturer’s instruction (MP Biomedical, Solon, OH, USA). Amplification of the V4 region was done using the primer 515F (5′-GTGYCAGCMGCCGCGGTA-3′)/806R (5′-GGACTACVSGGGTATCTAAT-3′), the bar-coded amplicons from each sample pooled together with equimolar concentrations and sequenced by an Illumina MiSeq platform. Primer sequences of the paired reads and the bases with low Phred scores at the ends of the reads were removed using Cutadapt^58^, applying the following parameters: -e 0.15 –m 100 –trim-n –discard-untrimmed –q 15,10. The paired-end reads were assembled and denoised by the dada2 plugin^59^ in QIIME2^60^; chimera sequences were removed and unique sequences clustered by the same plugin. Contamination related to the mitochondria, chloroplast, and cyanobacteria were removed by QIIME2 taxa filter-table and filter-seqs methods using the SILVA 132 database taxonomy assignment in feature-classifier plugin. The OTU representative sequences were then clustered at 100% similarity, and their taxonomy was assigned using the SILVA 132 database via QIIME2 classify-sklearn command with default settings. The relative abundances of the OTUs in each sample were used to generate correlation matrices for visualizing interactions between different taxa. OTUs unclassified at the generic level were not considered for generating the co-occurrence network analysis. Pairwise Spearman correlations, R score, and *p*-value were calculated between these OTUs using the Hmisc package version 4.1.1, and only OTUs with strong Spearman’s correlation coefficient (R > |± 0.6| at *p* < 0.05) were considered. These correlations were visualized using igraph package version 1.2.1.

Each node (or vertex) in the network represents an OTU (or a genus), and each edge indicates a strong and significant correlation. All analyses were performed in R version 3.5.0. The degree of centrality of each node was measured to determine the importance of nodes in the network (Fig. 2b). Accordingly, all the nodes were classified into three categories based on the abundance of links with other members in the network: key nodes (vertexes with the top 20% centrality), peripheral nodes (vertexes with last 20% centrality), and moderate nodes (rest of the vertexes). All analyses were performed by using psych package version 1.8.4.

### Determination of growth-promoting isolates

Key-node bacteria in the network were treated as the potential growth-promoting microorganisms and peripheral node as the indicator bacterium. Here, we have selected a *Chloroflexus islandicus* strain SYSU G100190R as the indicator bacterium. This strain was revived in an R2A broth, and streaked into a single colony on the R2A agar plate. Representative growth-promoting strains present in the key nodes of the network were cross-streaked on the same plate, and kept incubated at 55 °C under dark conditions unless otherwise mentioned. The growth of the indicator bacterium was observed every 24 h by observing for possible growth of any biofilm on the culture plates.

### Spent-culture medium preparation

The selected growth-promoting strain (*Tepidimonas* sp. strain **190W**) was grown into a conical flask containing R2A broth under shaking condition (55 °C, 180 rpm, dark). After attainment of the stationary growth phase, the supernatant and cell fractions were separated by centrifugation (10,000 × *g*, 10 min). The cells were re-suspended in 10 ml of PBS buffer (pH 8.0) and disrupted by ultrasonication (Sonics VC751, USA). The resulting lysates were centrifuged at 12,000 × *g* for 30 min at 4 °C. The cell-free extracts (cell lysate) and supernatant were filter-sterilized (0.22 μm, Millipore, USA) and added to the R2A agar. The efficacy of the various media supplements was determined by observing the growth of the indicator strain (*Chloroflexus* sp. strain **190R**) on R2A agar, R2A + 20%/10%/5%/1% supernatant and R2A + cell-free extract following 3-day incubation at 55 °C under aerobic and dark conditions.

The optimal growth enhancement of the selected supplement was further determined from a time-course biomass assay of the indicator *Chloroflexus* sp. **190R** incubated with supernatant harvested at different time intervals. For this experiment, the growth curve of the *Tepidimonas* sp. strain **190W** was determined by measuring the optical density (590 nm) of the cells grown in R2A broth for 0 h, 4 h, 8 h, 12 h, and 24 h at 55 °C. Simultaneously, a series of R2A broth media were prepared by supplementing with 10% spent-culture supernatants of *Tepidimonas* sp. **190W** harvested from 0 h, 4 h, 8 h, 12 h, and 24 h cultures. To these media, 1 mL aliquots of the indicator strain *Chloroflexus* sp. **190R** were incubated (3 days, 55 °C, 180 rpm, dark). Cell concentrations were calculated by measuring the optical density (OD) at 590 nm using microplate reader (Synergy H1, BioTek Instruments, Inc, USA). All the experiments were conducted with three repeats. The optimal harvesting period of the 10% spent-culture supernatant was finally selected for preparation of the SCM based on the observed biomass production. The basal isolation media for SCM preparation for the present study is R2A agar.

### Isolation using the standard and network-directed methods

Traditional isolation method included serial dilution plating on R2A, CC, T5, and *Thermus* 162 media (Supplementary Table 3), and incubation at 55 °C under aerobic and dark conditions for 1 week. Routine culturing of the purified strains was done on R2A medium at 45 °C. The strains were also maintained as cells suspension in glycerol (20%, w/v) at −80 °C for further use. Network-directed isolation method (referred hitherto as SCM isolation) (Fig. 1c) involved serial dilution plating on SCM (R2A + 24 h 10% spent-culture supernatant of *Tepidimonas* sp. **190W**).

### DNA extraction, 16S rRNA gene PCR amplification, sequencing, and identification

Genomic DNA of isolates was extracted by heating a loopfull of cultured bacteria cells in about 5-volume of 10% Chelex 100 (Bio-Rad, USA) suspension at 100 °C for 30 min^61^. PCR amplification of the 16S rRNA gene were done using the primer pair PA-PB (Sangon Biotech Co., Ltd., Shanghai, China) in a thermal cycler set with the following program: initial denaturation for 5 min at 94 °C, 30 cycles of amplification involving denaturation (94 °C, 45 s), annealing (56 °C, 30 s) and extension (72 °C, 90 s), and a final extension step at 72 °C for 10 min^62^. The amplicon was purified using a PCR purification kit (Sangon Biotech Co.). Sequencing was performed in a single direction using universal 16S rRNA primers. Strains showing sequence identities below 95.3% were selected and cloned to generate almost-complete sequences. All sequencing was carried out at Sangon Biotech Co., Ltd. (Shanghai, China).

Identity to validly described species was identified using the EzBioCloud database (https://www.ezbiocloud.net/). Previously uncultured isolates were also compared with published uncultured gene sequences via the nucleotide BLAST search at NCBI (https://www.ncbi.nlm.nih.gov/). Candidates for novel species were temporarily defined by sequence similarity threshold of 98.7%^63^. Similarly, candidate for novel genus and higher taxa were categorized using the 16S rRNA gene sequence identity threshold of 95.3- 90.0% and <90%, respectively^64^.

### Phylogenetic analysis

Partial 16S rRNA gene sequences (~800 bp) were aligned using CLUSTAL_X^65^. Gaps at the 5′ and 3′ ends of the alignment were manually removed. Phylogenetic analyses applying maximum-likelihood algorithm were performed using IQ-TREE version 1.6.10^66^, with the parameter “-alrt 1000 -bb 1000”. The topology of the phylogenetic trees was evaluated by the bootstrap resampling with 1000 replicates. The phylogeny of the isolates with lower similarities (<95.3%) was generated with the almost-complete 16S rRNA gene sequence in the pre-aligned tree using ARB database^67^.

### Comparative genomics analysis

Eight draft genomes of the genera *Tepidiomonas* and *Chloroflexus* were retrieved from the JGI database (https://jgi.doe.gov/). Functional annotations of the genes within these genomes were performed using TIGRfam and the DOE-JGI microbial genome annotation^68^. Statistical analyses of *p*-value and functional gene categories were calculated with ggpubr version 0.2 and ggplot2 version 2.2.1 in R version 3.5.0, respectively.

### Non-targeted metabolomics analysis of extracellular secretions

For extraction of the metabolites in the spent medium supernatant, 100-μL bacterial culture broth was taken into a 1.5-mL Eppendorf tube and centrifuged (14,000 × *g*, 20 min, 4 °C). The supernatant was transferred to a fresh 1.5-mL Eppendorf tube. Finally, four volumes of pre-chilled methanol were added, gently mixed, and incubated overnight at −80 °C. The mixture was centrifuged at 14,000 × *g* for 10 min at 4 °C, and the resulting supernatant transferred to a fresh Eppendorf tube. The pellet was dried using liquid N_2_, and stored in −80 °C freezer until further use.

The non-targeted metabolomics analysis was performed using Q Exactive Orbitrap LC-MS (Thermo, CA). One microliter of the supernatant was loaded to a normal phase chromatography column, and the sample eluted to an orbitrap mass spectrometer with 50% ACN containing 10 mM ammonium formate as eluent. Data with mass ranges of m/z 80-1200 and m/z 70-1050 were acquired at positive ion mode and negative ion mode, respectively, with data-dependent MS-MS acquisition. The full scan and fragment spectra were collected with a resolution of 70,000 and 17,500, respectively. The source parameters were: spray voltage: 3000 V; capillary temperature: 320 °C; heater temperature: 300 °C; sheath gas flow rate: 35; auxiliary gas flow rate: 10. Metabolite identification was based on Trace finder search with the home-built database. Metabolomic data analyses were then performed using MetaboAnalyst 3.0^69^.

### Growth-promotion assay

List of the selected fifteen compounds (metabolites) and the concentration of each of these metabolites for growth-promotion assay are listed in Supplementary Table 6. All compounds in analytical grade are procured from Aladdin Chemical Reagent Co. (China), except isopentenyladenine and hydroxypyruvate which are from Sigma. For the assay, 10-μL cell suspension of each of the representative strain grown in SCM-R2A broth was collected, centrifuged, and washed. These cells were inoculated into a 24-well culture plate (ThermoFisher), each well containing 1 mL R2A broth supplemented with one of the tested compounds. One well containing 1 mL of R2A broth without any compound was used as the negative control and another with 1 mL of SCM-R2A broth as positive control. The plate was incubated in an aerobic chamber, and kept incubated at 45 °C or 55 °C for 1 week. Cell concentrations were measured by observing the optical density (OD) at 590 nm using a microplate reader (Synergy H1, BioTek Instruments, Inc, USA). All the experiments were conducted with three repeats. Statistical analyses were calculated with tidyr version 0.8.3 and ggplot2 version 2.2.1 in R version 3.5.0.

## Supplementary information

Supplementary Information

Reporting Summary

## Data Availability

Raw sequences from the 16S Illumina sequencing of the Tibet hot spring samples are deposited at the Sequence Read Archive (SRP101393) in the NCBI under the BioProject accession no. PRJNA562830. The 16S rRNA gene sequences of the putative novel taxa isolated during the study are deposited in DDBJ/EMBL/GenBank databases under accession numbers MK714221-MK714339.
