# Supplementary material for: Network-directed efficient isolation of previously uncultivated Chloroflexi and related bacteria in hot spring microbial mats
Source: NPJ Biofilms Microbiomes. 2020 Apr 29;6:20. doi: 10.1038/s41522-020-0131-4 (PMC7190741; doi:10.1038/s41522-020-0131-4)
Supplement: Supplementary file 1 — Supplementary Information [file 41522_2020_131_MOESM1_ESM.pdf]

Supplementary Information for

Network-directed efficient isolation of previously uncultivated *Chloroflexi* and related bacteria in hot spring microbial mats

Wen-Dong Xian[1,†], Nimaichand Salam[1,†], Meng-Meng Li[1,†], En-Min Zhou[2], Yi-Rui Yin[1], Ze-Tao Liu[1], Yu-Zhen Ming[1], Xiao-Tong Zhang[1], Geng Wu[3], Lan Liu[1], Min Xiao[1], Hong-Chen Jiang[3] and Wen-Jun Li[1,*]

[1]State Key Laboratory of Biocontrol, Guangdong Provincial Key Laboratory of Plant Resources and Southern Marine Science and Engineering Guangdong Laboratory (Zhuhai), School of Life Sciences, Sun Yat-sen University, Guangzhou 510275, China

[2]School of Resource Environment and Earth Science, Yunnan Institute of Geography, Yunnan University, Kunming 650091, China

[3]State Key Laboratory of Biogeology and Environmental Geology, China University of Geosciences, Wuhan 430074, China.

*** Corresponding author:**

Wen-Jun Li, Email: liwenjun3@mail.sysu.edu.cn; Tel. and Fax: +86 20 8411 1727

[†] These authors contributed equally to this work.

Supplementary Table 1. Physicochemical parameters of the hot spring samples used in the study.

Sample	Location	ºN	ºE	Elevation	Temperature (ºC)	pH	Sampling time
S51	Tibet (QZM)	28.24646	91.79549	4388	63.0	6.8	2015.8
S52	Tibet (QZM)	28.24860	91.80365	4450	62.0	6.5	2015.8
S56	Tibet (QZM)	28.24816	91.80855	4505	67.0	7.0	2015.8
S57	Tibet (QZM)	28.24816	91.80855	4505	65.0	7.0	2015.8
S58	Tibet (QZM)	28.24816	91.80855	4505	64.0	6.8	2015.8
S59	Tibet (QZM)	28.24816	91.80855	4505	62.0	6.8	2015.8
S60	Tibet (QZM)	28.24851	91.80342	4438	65.7	6.7	2015.8
S61	Tibet (QZM)	28.24722	91.80970	4502	67.5	6.5	2015.8
S81	Tibet (QC)	30.66632	91.59135	4497	61.4	6.9	2016.8
S93	Tibet (DGJ)	29.60516	85.74628	5079	66.7	7.4	2016.8
S94	Tibet (DGJ)	29.60516	85.74628	5079	62.3	7.3	2016.8
Y1	Yunnan (Rehai)	24.94670	98.43709	1439	66.0	7.5	2017.5
Y2	Yunnan (Rehai)	24.94664	98.43703	1439	63.0	7.5	2017.5
Y3	Yunnan (Rehai)	24.94647	98.43682	1352	55.0	7.0	2017.5
A6	Tibet (DGQ)	82.10560	30.70526	4861	57.0	7.0	2018.8
A8	Tibet (DGQ)	82.10560	30.70526	4861	65.0	7.0	2018.8
A9	Tibet (DGQ)	82.10560	30.70526	4861	55.0	7.0	2018.8
A47	Tibet (MS)	80.75408	31.12466	4355	71.7	7.9	2018.8
A62	Tibet (MLJ)	80.49134	31.36898	4865	55.0	7.0	2018.8
A66	Tibet (BAR)	80.40778	31.44331	4807	55.0	6.5	2018.8
A75	Tibet (LJ)	80.36404	32.35644	4495	52.0	7.0	2018.8
A93	Tibet (DGJ)	29.59870	85.75069	5057	60.0	8.0	2018.8
A94	Tibet (DGJ)	29.59872	85.75070	5057	54.0	8.0	2018.8
A95	Tibet (DGJ)	29.59875	85.75074	5057	52.0	8.0	2018.8
A96	Tibet (DGJ)	29.59877	85.75076	5057	45.0	8.0	2018.8
A99	Tibet (DGJ)	29.60607	85.74900	5076	68.0	8.0	2018.8

Note: The samples Y1, Y2, Y3 and A96 were used for isolation.

Supplementary Table 2. Statistical parameters of the high-throughput sequencing.

Sample ID	Raw reads count	Filtered reads count	OTU count
S51	166889	140817	379
S52	100172	75934	310
S56	129194	101748	612
S57	113332	90315	633
S58	104825	83742	650
S59	118868	91745	722
S60	122801	99326	655
S61	149358	112642	537
S81	64946	46773	597
S93	114633	102512	316
S94	76605	69993	238
Y1	80448	74969	596
Y2	66516	62275	416
Y3	76393	70332	177
A6	105460	81111	511
A8	91599	74289	670
A9	118359	102334	518
A47	95561	71279	359
A62	96497	79830	340
A66	95559	74829	913
A75	113624	98565	199
A93	83907	67820	361
A94	99329	77926	342
A95	84620	70335	465
A96	91067	76169	385
A99	96662	76262	494

Supplementary Table 3. Conventional isolation media used in the current study.

Media	Composition (g/L)
Reasoner's 2A (R2A) agar<br>[DSM 830 medium]	Yeast extract 0.5, peptone 0.5, casamino acids 0.5, glucose 0.5, starch 0.5, K_2HPO_4 0.3, $MgSO_4$ 0.05, sodium pyruvate 0.3, agar 20.0, pH 7.2
CC agar	Microcrystalline cellulose 1.0, casamino acids 1.0, KNO_3 0.2, Na_2HPO_4 0.5, $MgSO_4 \cdot 7H_2O$ 0.05, $FeSO_4 \cdot 7H_2O$ 0.01, agar 20.0, pH 7.5<br>*Cellulose is one of the important carbon source in hot spring microbial mats.
Thermus 162 agar medium<br>(DSM 878 medium)	Yeast extract 1.0, tryptone 1.0, nitrilotriacetic acid 100.0 mg, $CaSO_4 \cdot 2H_2O$ 40.0 mg, $MgCl_2 \cdot 6H_2O$ 200.0 mg, 0.01 M ferric citrate 0.5 mL, trace element solution 0.5 mL, phosphate buffer 100.0 mL, agar 20.0, pH 7.2.<br>1. Phosphate buffer (g/L) [KH_2PO_4 5.44, $Na_2HPO_4 \cdot 12H_20$ 43.0, pH 7.2].<br>2. Trace element solution (g/L) [$MnSO_4 \cdot H_2O$ 2.28, $ZnSO_4 \cdot 7H_2O$ 0.5, H_3BO_3 0.5, $CuSO_4 \cdot 5H_2O$ $Na_2MoO_4 \cdot 2H_2O$ 25.0 mg, $CoCl_2 \cdot 6H_2O$ 45.0 mg]
T5 agar	Tryptone 0.5, yeast extract 2.0, glucose 1.0, lotus root starch 1.0, agar 20.0, pH 7.2

***Reference:**

Romling, U., and M. Y. Galperin (2015). Bacterial cellulose biosynthesis: diversity of operons, subunits, products, and functions. *Trends Microbiol* **23**: 545-557.

Supplementary Table 4. Taxonomic affiliation of the strains isolated in this study.

Strain ID	Sample	Isolation media	Group	Top-hit taxon	Top-hit strain	Similarity (%)
**SYSU GT96-39**	A96	SCM-R2A	Group 21	FJ529921	NBDTU1	89.23
**SYSU GT96-1**	A96	SCM-R2A	Group 18	*Schleiferia thermophila*	TU-20(T)	91.34
**SYSU GT96-37**	A96	SCM-R2A	Group 18	*Schleiferia thermophila*	TU-20(T)	91.34
**SYSU GT96-40-1**	A96	SCM-R2A	Group 20	AF445665	SM1C08	94.07
**SYSU GT96-46**	A96	SCM-R2A	Group 37	*Sandaracinobacter sibiricus*	RB16-17(T)	95.46
**SYSU GT96-18**	A96	R2A	Group 37	*Sandaracinobacter sibiricus*	RB16-17(T)	95.47
**SYSU GT96-61**	A96	R2A	Group 37	*Sandaracinobacter sibiricus*	RB16-17(T)	95.47
**SYSU GT96-4**	A96	SCM-R2A	Group 37	*Sandaracinobacter sibiricus*	RB16-17(T)	95.47
**SYSU GT96-72**	A96	R2A	Group 37	*Sandaracinobacter sibiricus*	RB16-17(T)	95.48
**SYSU GT96-53**	A96	SCM-R2A	Group 37	*Sandaracinobacter sibiricus*	RB16-17(T)	95.49
**SYSU GT96-54**	A96	SCM-R2A	Group 37	*Sandaracinobacter sibiricus*	RB16-17(T)	95.5
**SYSU GT96-34**	A96	SCM-R2A	Group 37	*Sandaracinobacter sibiricus*	RB16-17(T)	95.52
**SYSU GT96-47**	A96	SCM-R2A	Group 37	*Sandaracinobacter sibiricus*	RB16-17(T)	95.52
**SYSU GT96-57**	A96	SCM-R2A	Group 37	*Sandaracinobacter sibiricus*	RB16-17(T)	95.53
**SYSU GT96-56**	A96	SCM-R2A	Group 37	*Sandaracinobacter sibiricus*	RB16-17(T)	95.54
**SYSU GT96-71**	A96	R2A	Group 37	*Sandaracinobacter sibiricus*	RB16-17(T)	95.55
**SYSU GT96-34-1**	A96	SCM-R2A	Group 25	AF407720	B35	96.96
**SYSU GT96-19**	A96	R2A	Group 15	*Chloroflexus islandicus*	isl-2(T)	98.06
**SYSU GT96-45**	A96	SCM-R2A	Group 15	*Chloroflexus islandicus*	isl-2(T)	98.08
**SYSU GT96-3**	A96	SCM-R2A	Group 15	*Chloroflexus islandicus*	isl-2(T)	98.19
**SYSU GT96-40**	A96	SCM-R2A	Group 15	*Chloroflexus islandicus*	isl-2(T)	98.19
**SYSU GT96-52**	A96	SCM-R2A	Group 15	*Chloroflexus islandicus*	isl-2(T)	98.19
**SYSU GT96-20**	A96	R2A	Group 15	*Chloroflexus islandicus*	isl-2(T)	98.21
**SYSU GT96-17**	A96	R2A	Group 41	AM777983	CVCloAm2Ph106	98.74

**SYSU GT96-60**	A96	R2A	Group 39	*Elioraea tepidiphila*	DSM 17972(T)	99.74
**SYSU GT96-24**	A96	R2A	Group 39	*Elioraea tepidiphila*	DSM 17972(T)	99.75
**SYSU GT96-14**	A96	SCM-R2A	Group 19	*Roseomonas alkaliterrae*	YIM 78007(T)	99.87
**SYSU GT96-26**	A96	R2A	Group 38	LJIY	FJAT-21963	100
**SYSU GT96-12**	A96	SCM-R2A	Group 38	LJIY	FJAT-21963	100
**SYSU GY1-61**	Y1	SCM-*Thermus* 162 medium	Group 8	JN825371	Alchichica_Al52_2_1B_100	89.81
**SYSU GY1-62**	Y1	SCM-*Thermus* 162 medium	Group 8	JN825371	Alchichica_Al52_2_1B_100	89.81
**SYSU GY1-30**	Y1	SCM-R2A	Group 18	*Schleiferia thermophila*	TU-20(T)	92.99
**SYSU GY1-233**	Y1	*Thermus* 162 medium	Group 36	DQ129304	AKIW813	93.42
**SYSU GY1-259**	Y1	SCM-*Thermus* 162 medium	Group 36	DQ129304	AKIW813	93.42
**SYSU GY1-P12**	Y1	SCM-R2A	Group 36	EF516779	FCPP721	93.87
**SYSU GY1-29**	Y1	SCM-R2A	Group 14	*Chloroflexus islandicus*	isl-2(T)	94.74
**SYSU GY1-23**	Y1	SCM-R2A	Group 36	EF205457	YCB86	94.99
**SYSU GY1-75**	Y1	R2A	Group 37	*Sandaracinobacter sibiricus*	RB16-17(T)	95.47
**SYSU GY1-100**	Y1	R2A	Group 27	JQ426256	AN0C1AD06	95.53
**SYSU GY1-247**	Y1	*Thermus* 162 medium	Group 26	*Meiothermus cateniformans*	LY1(T)	95.57
**SYSU GY1-96**	Y1	R2A	Group 27	JN038861	P-R74	95.71
**SYSU GY1-59**	Y1	SCM-*Thermus* 162 medium	Group 29	*Thermus tengchongensis*	YIM 77924(T)	96.3
**SYSU GY1-65**	Y1	SCM-*Thermus* 162 medium	Group 13	*Roseiflexus castenholzii*	DSM 13941(T)	96.52
**SYSU GY1-4**	Y1	SCM-R2A	Group 12	*Tepidimonas ignava*	SPS-1037(T)	96.68
**SYSU GY1-28**	Y1	SCM-R2A	Group 12	*Tepidimonas ignava*	SPS-1037(T)	96.92
**SYSU GY1-58**	Y1	SCM-*Thermus* 162 medium	Group 12	*Tepidimonas ignava*	SPS-1037(T)	96.92
**SYSU GY1-64**	Y1	SCM-*Thermus* 162 medium	Group 12	*Tepidimonas ignava*	SPS-1037(T)	97
**SYSU GY1-60**	Y1	SCM-*Thermus* 162 medium	Group 12	*Tepidimonas ignava*	SPS-1037(T)	97.04
**SYSU GY1-63**	Y1	SCM-*Thermus* 162 medium	Group 12	*Tepidimonas ignava*	SPS-1037(T)	97.08
**SYSU GY1-278**	Y1	SCM-*Thermus* 162 medium	Group 25	AF407725	B63	97.23
**SYSU GY1-272**	Y1	SCM-*Thermus* 162 medium	Group 25	AF407725	B63	97.27
**SYSU GY1-174**	Y1	SCM-R2A	Group 26	*Meiothermus hypogaeus*	AZM34c11(T)	97.38

**SYSU GY1-105**	Y1	R2A	Group 25	AF407720	B35	97.76
**SYSU GY1-82**	Y1	R2A	Group 25	AF407720	B35	97.77
**SYSU GY1-241**	Y1	*Thermus* 162 medium	Group 25	AF407720	B35	97.78
**SYSU GY1-224**	Y1	*Thermus* 162 medium	Group 25	AF407720	B35	97.79
**SYSU GY1-66**	Y1	SCM-*Thermus* 162 medium	Group 12	*Tepidimonas ignava*	SPS-1037(T)	97.81
**SYSU GY1-56**	Y1	SCM-*Thermus* 162 medium	Group 12	*Tepidimonas ignava*	SPS-1037(T)	97.83
**SYSU GY1-67**	Y1	SCM-*Thermus* 162 medium	Group 12	*Tepidimonas ignava*	SPS-1037(T)	97.84
**SYSU GY1-57**	Y1	SCM-*Thermus* 162 medium	Group 12	*Tepidimonas ignava*	SPS-1037(T)	97.92
**SYSU GY1-239**	Y1	*Thermus* 162 medium	Group 33	AF407714	B11	98.17
**SYSU GY1-242**	Y1	*Thermus* 162 medium	Group 33	AF407714	B11	98.17
**SYSU GY1-67P**	Y1	SCM-*Thermus* 162 medium	Group 12	*Tepidimonas ignava*	SPS-1037(T)	98.17
**SYSU GY1-38P**	Y1	SCM-R2A	Group 23	*Thermus calditerrae*	YIM 77925(T)	98.18
**SYSU GY1-99**	Y1	R2A	Group 23	*Thermus calditerrae*	YIM 77925(T)	98.2
**SYSU GY1-170**	Y1	SCM-R2A	Group 26	*Meiothermus cerbereus*	DSM 11376(T)	98.23
**SYSU GY1-122**	Y1	SCM-R2A	Group 23	*Thermus calditerrae*	YIM 77925(T)	98.28
**SYSU GY1-284**	Y1	SCM-*Thermus* 162 medium	Group 26	*Meiothermus cerbereus*	DSM 11376(T)	98.3
**SYSU GY1-252**	Y1	SCM-*Thermus* 162 medium	Group 23	*Thermus calditerrae*	YIM 77925(T)	98.3
**SYSU GY1-283**	Y1	SCM-*Thermus* 162 medium	Group 23	*Thermus calditerrae*	YIM 77925(T)	98.3
**SYSU GY1-222**	Y1	*Thermus* 162 medium	Group 26	*Meiothermus cerbereus*	DSM 11376(T)	98.31
**SYSU GY1-182**	Y1	SCM-R2A	Group 26	*Meiothermus cerbereus*	DSM 11376(T)	98.31
**SYSU GY1-253**	Y1	SCM-*Thermus* 162 medium	Group 26	*Meiothermus cerbereus*	DSM 11376(T)	98.31
**SYSU GY1-254**	Y1	SCM-*Thermus* 162 medium	Group 26	*Meiothermus cerbereus*	DSM 11376(T)	98.31
**SYSU GY1-277**	Y1	SCM-*Thermus* 162 medium	Group 23	*Thermus calditerrae*	YIM 77925(T)	98.31
**SYSU GY1-257**	Y1	SCM-*Thermus* 162 medium	Group 26	*Meiothermus cerbereus*	DSM 11376(T)	98.32
**SYSU GY1-258**	Y1	SCM-*Thermus* 162 medium	Group 26	*Meiothermus cerbereus*	DSM 11376(T)	98.32
**SYSU GY1-164**	Y1	SCM-R2A	Group 26	*Meiothermus cerbereus*	DSM 11376(T)	98.35
**SYSU GY1-5**	Y1	SCM-R2A	Group 40	*Chloroflexus islandicus*	isl-2(T)	98.67
**SYSU GY1-P29**	Y1	SCM-R2A	Group 2	*Thermoleophilum album*	ATCC 35263(T)	98.7

**SYSU GY1-83**	Y1	R2A	Group 41	AM777983	CVCloAm2Ph106	98.7
**SYSU GY1-86**	Y1	R2A	Group 41	AM777983	CVCloAm2Ph106	98.7
**SYSU GY1-106**	Y1	R2A	Group 41	AM777983	CVCloAm2Ph106	98.7
**SYSU GY1-236**	Y1	*Thermus* 162 medium	Group 41	AM777983	CVCloAm2Ph106	98.7
**SYSU GY1-141**	Y1	SCM-R2A	Group 41	AM777983	CVCloAm2Ph106	98.7
**SYSU GY1-87**	Y1	R2A	Group 41	AM777983	CVCloAm2Ph106	98.71
**SYSU GY1-149**	Y1	SCM-R2A	Group 41	AM777983	CVCloAm2Ph106	98.71
**SYSU GY1-1**	Y1	SCM-R2A	Group 41	AM777983	CVCloAm2Ph106	98.72
**SYSU GY1-251**	Y1	SCM-*Thermus* 162 medium	Group 23	*Thermus caliditerrae*	YIM 77925(T)	98.95
**SYSU GY1-184**	Y1	SCM-R2A	Group 40	*Chloroflexus islandicus*	isl-2(T)	99.04
**SYSU GY1-21**	Y1	SCM-R2A	Group 40	*Chloroflexus islandicus*	isl-2(T)	99.08
**SYSU GY1-140**	Y1	SCM-R2A	Group 40	*Chloroflexus islandicus*	isl-2(T)	99.08
**SYSU GY1-159**	Y1	SCM-R2A	Group 40	*Chloroflexus islandicus*	isl-2(T)	99.08
**SYSU GY1-212**	Y1	SCM-R2A	Group 40	*Chloroflexus islandicus*	isl-2(T)	99.08
**SYSU GY1-183**	Y1	SCM-R2A	Group 40	*Chloroflexus islandicus*	isl-2(T)	99.09
**SYSU GY1-77**	Y1	R2A	Group 40	*Chloroflexus islandicus*	isl-2(T)	99.1
**SYSU GY1-160**	Y1	SCM-R2A	Group 40	*Chloroflexus islandicus*	isl-2(T)	99.14
**SYSU GY1-158**	Y1	SCM-R2A	Group 40	*Chloroflexus islandicus*	isl-2(T)	99.21
**SYSU GY1-195**	Y1	SCM-R2A	Group 40	*Chloroflexus islandicus*	isl-2(T)	99.22
**SYSU GY1-272-2**	Y1	SCM-*Thermus* 162 medium	Group 40	*Chloroflexus islandicus*	isl-2(T)	99.22
**SYSU GY1-71**	Y1	SCM-*Thermus* 162 medium	Group 35	AY753393	SK47	99.23
**SYSU GY1-179**	Y1	SCM-R2A	Group 6	*Francisella piscicida*	GM2212(T)	99.25
**SYSU GY1-103**	Y1	SCM-R2A	Group 35	AY753393	SK47	99.32
**SYSU GY1-84**	Y1	SCM-R2A	Group 35	AY753393	SK47	99.33
**SYSU GY1-11**	Y1	SCM-R2A	Group 40	*Chloroflexus islandicus*	isl-2(T)	99.35
**SYSU GY1-12**	Y1	SCM-R2A	Group 40	*Chloroflexus islandicus*	isl-2(T)	99.35
**SYSU GY1-121**	Y1	SCM-R2A	Group 40	*Chloroflexus islandicus*	isl-2(T)	99.35
**SYSU GY1-146**	Y1	SCM-R2A	Group 40	*Chloroflexus islandicus*	isl-2(T)	99.35

**SYSU GY1-22**	Y1	SCM-R2A	Group 35	AY753393	SK47	99.35
**SYSU GY1-68**	Y1	SCM-*Thermus* 162 medium	Group 35	AY753393	SK47	99.35
**SYSU GY1-69**	Y1	SCM-*Thermus* 162 medium	Group 35	AY753393	SK47	99.35
**SYSU GY1-228**	Y1	*Thermus* 162 medium	Group 30	*Thermus caliditerrae*	YIM 77925(T)	99.35
**SYSU GY1-55**	Y1	SCM-*Thermus* 162 medium	Group 30	*Thermus caliditerrae*	YIM 77925(T)	99.35
**SYSU GY1-57-2**	Y1	SCM-*Thermus* 162 medium	Group 30	*Thermus caliditerrae*	YIM 77925(T)	99.35
**SYSU GY1-19**	Y1	SCM-R2A	Group 35	AY753393	SK47	99.36
**SYSU GY1-156**	Y1	SCM-R2A	Group 40	*Chloroflexus islandicus*	isl-2(T)	99.37
**SYSU GY1-157**	Y1	SCM-R2A	Group 40	*Chloroflexus islandicus*	isl-2(T)	99.37
**SYSU GY1-109**	Y1	SCM-R2A	Group 35	AY753393	SK47	99.37
**SYSU GY1-80**	Y1	R2A	Group 28	AY555804	PK324	99.38
**SYSU GY1-89**	Y1	R2A	Group 40	*Chloroflexus islandicus*	isl-2(T)	99.48
**SYSU GY1-178**	Y1	SCM-R2A	Group 40	*Chloroflexus islandicus*	isl-2(T)	99.49
**SYSU GY1-74**	Y1	R2A	Group 39	*Elioraea tepidiphila*	DSM 17972(T)	99.74
**SYSU GYI-238-2**	Y1	*Thermus* 162 medium	Group 22	*Thiobacter subterraneus*	NCTC9001(T)	99.74
**SYSU GY1-173**	Y1	SCM-R2A	Group 29	*Thermus tengchongensis*	YIM 77924(T)	99.87
**SYSU GY1-136**	Y1	SCM-R2A	Group 9	*Bacillus siamensis*	KCTC 13613(T)	99.87
**SYSU GY1-139**	Y1	SCM-R2A	Group 9	*Bacillus siamensis*	KCTC 13613(T)	99.87
**SYSU GY1-176**	Y1	SCM-R2A	Group 9	*Bacillus siamensis*	KCTC 13613(T)	99.87
**SYSU GY1-273**	Y1	SCM-*Thermus* 162 medium	Group 9	*Bacillus siamensis*	KCTC 13613(T)	99.87
**SYSU GY1-35-1**	Y1	SCM-R2A	Group 7	*Caldimonas taiwanensis*	NBRC 104434(T)	99.87
**SYSU GY1-36**	Y1	SCM-R2A	Group 7	*Caldimonas taiwanensis*	NBRC 104434(T)	99.87
**SYSU GY1-37**	Y1	SCM-R2A	Group 7	*Caldimonas taiwanensis*	NBRC 104434(T)	99.87
**SYSU GY1-211**	Y1	SCM-R2A	Group 4	*Tepidimonas fonticaldi*	AT-A2(T)	99.87
**SYSU GY1-73**	Y1	SCM-*Thermus* 162 medium	Group 7	*Tepidimonas taiwanensis*	I1-1(T)	99.87
**SYSU GY1-226**	Y1	*Thermus* 162 medium	Group 29	*Thermus tengchongensis*	YIM 77924(T)	100
**SYSU GY1-243**	Y1	*Thermus* 162 medium	Group 29	*Thermus tengchongensis*	YIM 77924(T)	100
**SYSU GY1-128**	Y1	SCM-R2A	Group 29	*Thermus tengchongensis*	YIM 77924(T)	100

**SYSU GY1-175**	Y1	SCM-R2A	Group 29	*Thermus tengchongensis*	YIM 77924(T)	100
**SYSU GY1-181**	Y1	SCM-R2A	Group 29	*Thermus tengchongensis*	YIM 77924(T)	100
**SYSU GY1-78**	Y1	R2A	Group 24	*Thermus brockianus*	YS038(T)	100
**SYSU GY1-90**	Y1	R2A	Group 24	*Thermus brockianus*	YS038(T)	100
**SYSU GY1-91**	Y1	R2A	Group 24	*Thermus brockianus*	YS038(T)	100
**SYSU GY1-54**	Y1	SCM-*Thermus* 162 medium	Group 24	*Thermus brockianus*	YS038(T)	100
**SYSU GY1-276**	Y1	SCM-*Thermus* 162 medium	Group 24	*Thermus brockianus*	YS038(T)	100
**SYSU GY1-232**	Y1	*Thermus* 162 medium	Group 22	*Thiobacter subterraneus*	C55(T)	100
**SYSU GY1-234**	Y1	*Thermus* 162 medium	Group 22	*Thiobacter subterraneus*	C55(T)	100
**SYSU GY2-132**	Y2	SCM-R2A	Group 36	DQ129304	AKIW813	93.48
**SYSU GY2-90**	Y2	SCM-R2A	Group 13	*Roseiflexus castenholzii*	DSM 13941(T)	96.07
**SYSU GY2-8**	Y2	SCM-R2A	Group 13	*Roseiflexus castenholzii*	DSM 13941(T)	96.09
**SYSU GY2-10**	Y2	SCM-R2A	Group 13	*Roseiflexus castenholzii*	DSM 13941(T)	96.09
**SYSU GY2-99**	Y2	SCM-R2A	Group 13	*Roseiflexus castenholzii*	DSM 13941(T)	96.17
**SYSU GY2-6**	Y2	SCM-R2A	Group 13	*Roseiflexus castenholzii*	DSM 13941(T)	96.24
**SYSU GY2-106**	Y2	SCM-R2A	Group 10	AF521186	RH-914	96.35
**SYSU GY2-123**	Y2	SCM-R2A	Group 10	AF521186	RH-914	96.39
**SYSU GY2-124**	Y2	SCM-R2A	Group 10	AF521186	RH-914	96.39
**SYSU GY2-136**	Y2	SCM-R2A	Group 10	AF521186	RH-914	96.39
**SYSU GY2-110**	Y2	SCM-R2A	Group 10	AF521186	RH-914	96.46
**SYSU GY2-121**	Y2	SCM-R2A	Group 10	AF521186	RH-914	96.48
**SYSU GY2-86**	Y2	SCM-R2A	Group 10	AF521186	RH-914	96.49
**SYSU GY2-115**	Y2	SCM-R2A	Group 10	AF521186	RH-914	96.57
**SYSU GY2-32**	Y2	SCM-*Thermus* 162 medium	Group 12	*Tepidimonas ignava*	SPS-1037(T)	96.7
**SYSU GY2-128**	Y2	SCM-R2A	Group 25	AF407725	B63	96.95
**SYSU GY2-12**	Y2	SCM-R2A	Group 26	*Meiothermus hypogaeus*	AZM34c11(T)	97.43
**SYSU GY2-45**	Y2	SCM-*Thermus* 162 medium	Group 12	*Tepidimonas ignava*	SPS-1037(T)	98.18
**SYSU GY2-68**	Y2	SCM-*Thermus* 162 medium	Group 24	*Thermus brockianus*	YS038(T)	98.83

**SYSU GY2-54**	Y2	SCM-*Thermus* 162 medium	Group 35	AY753393	SK47	99.23
**SYSU GY2-42**	Y2	SCM-*Thermus* 162 medium	Group 29	*Thermus tengchongensis*	YIM 77924(T)	99.25
**SYSU GY2-29？**	Y2	SCM-*Thermus* 162 medium	Group 30	*Thermus caliditerrae*	YIM 77925(T)	99.35
**SYSU GY2-104**	Y2	SCM-R2A	Group 40	*Chloroflexus islandicus*	isl-2(T)	99.47
**SYSU GY2-88**	Y2	SCM-R2A	Group 40	*Chloroflexus islandicus*	isl-2(T)	99.48
**SYSU GY2-135**	Y2	SCM-R2A	Group 40	*Chloroflexus islandicus*	isl-2(T)	99.48
**SYSU GY2-39**	Y2	SCM-*Thermus* 162 medium	Group 29	*Thermus tengchongensis*	YIM 77924(T)	99.48
**SYSU GY2-83**	Y2	R2A	Group 40	*Chloroflexus islandicus*	isl-2(T)	99.51
**SYSU GY2-66**	Y2	SCM-Thermus 162 medium	Group 29	*Thermus tengchongensis*	YIM 77924(T)	99.61
**SYSU GY2-76**	Y2	*Thermus* 162 medium	Group 29	*Thermus tengchongensis*	YIM 77924(T)	99.74
**SYSU GY2-3**	Y2	SCM-R2A	Group 29	*Thermus tengchongensis*	YIM 77924(T)	99.74
**SYSU GY2-67**	Y2	SCM-*Thermus* 162 medium	Group 29	*Thermus tengchongensis*	YIM 77924(T)	99.74
**SYSU GY2-27**	Y2	SCM-*Thermus* 162 medium	Group 29	*Thermus tengchongensis*	YIM 77924(T)	99.76
**SYSU GY2-33**	Y2	SCM-*Thermus* 162 medium	Group 29	*Thermus tengchongensis*	YIM 77924(T)	99.85
**SYSU GY2-44**	Y2	SCM-*Thermus* 162 medium	Group 29	*Thermus tengchongensis*	YIM 77924(T)	99.86
**SYSU GY2-9**	Y2	SCM-R2A	Group 29	*Thermus tengchongensis*	YIM 77924(T)	99.87
**SYSU GY2-93**	Y2	SCM-R2A	Group 29	*Thermus tengchongensis*	YIM 77924(T)	99.87
**SYSU GY2-120**	Y2	SCM-R2A	Group 29	*Thermus tengchongensis*	YIM 77924(T)	99.87
**SYSU GY2-129**	Y2	SCM-R2A	Group 29	*Thermus tengchongensis*	YIM 77924(T)	99.87
**SYSU GY2-141**	Y2	SCM-R2A	Group 29	*Thermus tengchongensis*	YIM 77924(T)	99.87
**SYSU GY2-20**	Y2	SCM-*Thermus* 162 medium	Group 29	*Thermus tengchongensis*	YIM 77924(T)	99.87
**SYSU GY2-31**	Y2	SCM-*Thermus* 162 medium	Group 29	*Thermus tengchongensis*	YIM 77924(T)	99.87
**SYSU GY2-35**	Y2	SCM-*Thermus* 162 medium	Group 29	*Thermus tengchongensis*	YIM 77924(T)	99.87
**SYSU GY2-40**	Y2	SCM-*Thermus* 162 medium	Group 29	*Thermus tengchongensis*	YIM 77924(T)	99.87
**SYSU GY2-46**	Y2	SCM-*Thermus* 162 medium	Group 29	*Thermus tengchongensis*	YIM 77924(T)	99.87
**SYSU GY2-60**	Y2	SCM-*Thermus* 162 medium	Group 29	*Thermus tengchongensis*	YIM 77924(T)	99.87
**SYSU GY2-62**	Y2	SCM-*Thermus* 162 medium	Group 29	*Thermus tengchongensis*	YIM 77924(T)	99.87
**SYSU GY2-69**	Y2	SCM-*Thermus* 162 medium	Group 29	*Thermus tengchongensis*	YIM 77924(T)	99.87

**SYSU GY2-98**	Y2	SCM-R2A	Group 9	*Bacillus siamensis*	KCTC 13613(T)	99.87
**SYSU GY2-155**	Y2	SCM-R2A	Group 9	*Bacillus siamensis*	KCTC 13613(T)	99.87
**SYSU GY2-148**	Y2	SCM-R2A	Group 9	*Bacillus siamensis*	KCTC 13613(T)	99.88
**SYSU GY2-14**	Y2	*Thermus* 162 medium	Group 29	*Thermus tengchongensis*	YIM 77924(T)	100
**SYSU GY2-75**	Y2	*Thermus* 162 medium	Group 29	*Thermus tengchongensis*	YIM 77924(T)	100
**SYSU GY2-48**	Y2	SCM-*Thermus* 162 medium	Group 29	*Thermus tengchongensis*	YIM 77924(T)	100
**SYSU GY2-89**	Y2	SCM-R2A	Group 29	*Thermus tengchongensis*	YIM 77924(T)	100
**SYSU GY2-97**	Y2	SCM-R2A	Group 29	*Thermus tengchongensis*	YIM 77924(T)	100
**SYSU GY2-105**	Y2	SCM-R2A	Group 29	*Thermus tengchongensis*	YIM 77924(T)	100
**SYSU GY2-140**	Y2	SCM-R2A	Group 29	*Thermus tengchongensis*	YIM 77924(T)	100
**SYSU GY2-34**	Y2	SCM-*Thermus* 162 medium	Group 29	*Thermus tengchongensis*	YIM 77924(T)	100
**SYSU GY2-47**	Y2	SCM-*Thermus* 162 medium	Group 29	*Thermus tengchongensis*	YIM 77924(T)	100
**SYSU GY2-58**	Y2	SCM-*Thermus* 162 medium	Group 29	*Thermus tengchongensis*	YIM 77924(T)	100
**SYSU GY3-13**	Y3	SCM-R2A	Group 36	EF205457	YCB86	92.76
**SYSU GY3-101**	Y3	SCM-*Thermus* 162 medium	Group 15	*Chloroflexus islandicus*	isl-2(T)	93.39
**SYSU GY3-23**	Y3	SCM-R2A	Group 18	*Schleiferia thermophila*	TU-20(T)	93.43
**SYSU GY3-42**	Y3	R2A	Group 37	*Sandaracinobacter sibiricus*	RB16-17(T)	94.8
**SYSU GY3-136**	Y3	SCM-R2A	Group 37	*Sandaracinobacter sibiricus*	RB16-17(T)	94.93
**SYSU GY3-35**	Y3	SCM-R2A	Group 37	*Sandaracinobacter sibiricus*	RB16-17(T)	95.06
**SYSU GY3-37**	Y3	R2A	Group 37	*Sandaracinobacter sibiricus*	RB16-17(T)	95.07
**SYSU GY-37**	Y3	SCM-R2A	Group 37	*Sandaracinobacter sibiricus*	RB16-17(T)	95.19
**SYSU GY3-P8**	Y3	SCM-*Thermus* 162 medium	Group 13	*Roseiflexus castenholzii*	DSM 13941(T)	95.44
**SYSU GY3-138**	Y3	SCM-R2A	Group 13	*Roseiflexus castenholzii*	DSM 13941(T)	95.51
**SYSU GY3-144**	Y3	SCM-R2A	Group 36	EF205514	DTM38	95.69
**SYSU GY3-41**	Y3	R2A	Group 34	*Elioraea tepidiphila*	DSM 17972(T)	95.82
**SYSU GY3-147**	Y3	SCM-R2A	Group 10	AF521186	RH-914	96.12
**SYSU GY3-17**	Y3	SCM-R2A	Group 14	*Chloroflexus islandicus*	isl-2(T)	96.22
**SYSU GY3-112**	Y3	SCM-R2A	Group 10	AF521186	RH-914	96.38

**SYSU GY3-6-2**	Y3	SCM-R2A	Group 13	*Roseiflexus castenholzii*	DSM 13941(T)	96.39
**SYSU GY3-63**	Y3	SCM-*Thermus* 162 medium	Group 25	AF407725	B63	96.88
**SYSU GY3-141**	Y3	SCM-R2A	Group 25	AF407725	B63	97.15
**SYSU GY3-81**	Y3	*Thermus* 162 medium	Group 32	JX298759	9B-25	97.58
**SYSU GY3-83**	Y3	*Thermus* 162 medium	Group 32	JX298759	9B-25	97.79
**SYSU GY3-67**	Y3	SCM-*Thermus* 162 medium	Group 32	JX298759	9B-25	97.8
**SYSU GY3-146**	Y3	SCM-R2A	Group 23	*Thermus calidíterrae*	YIM 77925(T)	98.05
**SYSU GY3-201**	Y3	SCM-R2A	Group 23	*Thermus caliditerrae*	YIM 77925(T)	98.05
**SYSU GY3-61**	Y3	SCM-*Thermus* 162 medium	Group 12	*Tepidimonas ignava*	SPS-1037(T)	98.06
**SYSU GY3-77**	Y3	*Thermus* 162 medium	Group 33	AF407714	B11	98.18
**SYSU GY3-85**	Y3	*Thermus* 162 medium	Group 33	AF407714	B11	98.18
**SYSU GY3-193**	Y3	SCM-*Thermus* 162 medium	Group 23	*Thermus caliditerrae*	YIM 77925(T)	98.18
**SYSU GY3-150**	Y3	SCM-R2A	Group 23	*Thermus caliditerrae*	YIM 77925(T)	98.19
**SYSU GY3-89**	Y3	R2A	Group 36	EF205457	YCB86	98.3
**SYSU GY3-38**	Y3	R2A	Group 23	*Thermus caliditerrae*	YIM 77925(T)	98.31
**SYSU GY3-121**	Y3	SCM-R2A	Group 23	*Thermus caliditerrae*	YIM 77925(T)	98.32
**SYSU GY3-194**	Y3	SCM-*Thermus* 162 medium	Group 23	*Thermus caliditerrae*	YIM 77925(T)	98.32
**SYSU GY3-33**	Y3	SCM-R2A	Group 23	*Thermus caliditerrae*	YIM 77925(T)	98.34
**SYSU GY3-31**	Y3	SCM-R2A	Group 23	*Thermus caliditerrae*	YIM 77925(T)	98.7
**SYSU GY3-36**	Y3	SCM-R2A	Group 23	*Thermus caliditerrae*	YIM 77925(T)	98.7
**SYSU GY3-192**	Y3	SCM-*Thermus* 162 medium	Group 23	*Thermus caliditerrae*	YIM 77925(T)	98.7
**SYSU GY3-96**	Y3	SCM-*Thermus* 162 medium	Group 23	*Thermus caliditerrae*	YIM 77925(T)	98.88
**SYSU GY3-14**	Y3	SCM-R2A	Group 23	*Thermus caliditerrae*	YIM 77925(T)	98.96
**SYSU GY3-44**	Y3	R2A	Group 40	*Chloroflexus islandicus*	isl-2(T)	99.09
**SYSU GY3-188**	Y3	*Thermus* 162 medium	Group 30	*Thermus caliditerrae*	YIM 77925(T)	99.09
**SYSU GY3-196**	Y3	SCM-*Thermus* 162 medium	Group 30	*Thermus caliditerrae*	YIM 77925(T)	99.09
**SYSU GY3-197**	Y3	SCM-*Thermus* 162 medium	Group 30	*Thermus caliditerrae*	YIM 77925(T)	99.09
**SYSU GY3-199**	Y3	SCM-*Thermus* 162 medium	Group 30	*Thermus caliditerrae*	YIM 77925(T)	99.09

**SYSU GY3-19**	Y3	SCM-R2A	Group 35	AY753393	SK47	99.22
**SYSU GY3-20**	Y3	SCM-R2A	Group 35	AY753393	SK47	99.22
**SYSU GY3-116**	Y3	SCM-R2A	Group 30	*Thermus caliditerrae*	YIM 77925(T)	99.22
**SYSU GY3-59**	Y3	SCM-*Thermus* 162 medium	Group 30	*Thermus caliditerrae*	YIM 77925(T)	99.22
**SYSU GY3-191**	Y3	SCM-*Thermus* 162 medium	Group 30	*Thermus caliditerrae*	YIM 77925(T)	99.22
**SYSU GY3-195**	Y3	SCM-*Thermus* 162 medium	Group 30	*Thermus caliditerrae*	YIM 77925(T)	99.22
**SYSU GY3-102**	Y3	SCM-*Thermus* 162 medium	Group 30	*Thermus caliditerrae*	YIM 77925(T)	99.24
**SYSU GY3-142**	Y3	SCM-R2A	Group 35	AY753393	SK47	99.25
**SYSU GY3-15**	Y3	SCM-R2A	Group 30	*Thermus caliditerrae*	YIM 77925(T)	99.32
**SYSU GY3-49**	Y3	SCM-R2A	Group 35	AY753393	SK47	99.34
**SYSU GY3-52**	Y3	SCM-R2A	Group 35	AY753393	SK47	99.34
**SYSU GY3-53**	Y3	SCM-R2A	Group 35	AY753393	SK47	99.35
**SYSU GY3-24**	Y3	SCM-R2A	Group 35	AY753393	SK47	99.35
**SYSU GY3-143**	Y3	SCM-R2A	Group 35	AY753393	SK47	99.35
**SYSU GY3-97**	Y3	SCM-*Thermus* 162 medium	Group 29	*Thermus tengchongensis*	YIM 77924(T)	99.35
**SYSU GY3-39**	Y3	R2A	Group 30	*Thermus caliditerrae*	YIM 77925(T)	99.35
**SYSU GY3-40**	Y3	R2A	Group 30	*Thermus caliditerrae*	YIM 77925(T)	99.35
**SYSU GY3-41-2**	Y3	R2A	Group 30	*Thermus caliditerrae*	YIM 77925(T)	99.35
**SYSU GY3-46**	Y3	R2A	Group 30	*Thermus caliditerrae*	YIM 77925(T)	99.35
**SYSU GY3-47**	Y3	R2A	Group 30	*Thermus caliditerrae*	YIM 77925(T)	99.35
**SYSU GY3-50**	Y3	R2A	Group 30	*Thermus caliditerrae*	YIM 77925(T)	99.35
**SYSU GY3-51**	Y3	R2A	Group 30	*Thermus caliditerrae*	YIM 77925(T)	99.35
**SYSU GY3-54**	Y3	R2A	Group 30	*Thermus caliditerrae*	YIM 77925(T)	99.35
**SYSU GY3-55**	Y3	R2A	Group 30	*Thermus caliditerrae*	YIM 77925(T)	99.35
**SYSU GY3-56**	Y3	R2A	Group 30	*Thermus caliditerrae*	YIM 77925(T)	99.35
**SYSU GY3-86**	Y3	*Thermus* 162 medium	Group 30	*Thermus caliditerrae*	YIM 77925(T)	99.35
**SYSU GY3-18**	Y3	SCM-R2A	Group 30	*Thermus caliditerrae*	YIM 77925(T)	99.35
**SYSU GY3-30**	Y3	SCM-R2A	Group 30	*Thermus caliditerrae*	YIM 77925(T)	99.35

**SYSU GY3-32**	Y3	SCM-R2A	Group 30	*Thermus caliditerrae*	YIM 77925(T)	99.35
**SYSU GY3-118**	Y3	SCM-R2A	Group 30	*Thermus caliditerrae*	YIM 77925(T)	99.35
**SYSU GY3-120**	Y3	SCM-R2A	Group 30	*Thermus caliditerrae*	YIM 77925(T)	99.35
**SYSU GY3-126**	Y3	SCM-R2A	Group 30	*Thermus caliditerrae*	YIM 77925(T)	99.35
**SYSU GY3-129**	Y3	SCM-R2A	Group 30	*Thermus caliditerrae*	YIM 77925(T)	99.35
**SYSU GY3-57**	Y3	SCM-*Thermus* 162 medium	Group 30	*Thermus caliditerrae*	YIM 77925(T)	99.35
**SYSU GY3-60**	Y3	SCM-*Thermus* 162 medium	Group 30	*Thermus caliditerrae*	YIM 77925(T)	99.35
**SYSU GY3-62**	Y3	SCM-*Thermus* 162 medium	Group 30	*Thermus caliditerrae*	YIM 77925(T)	99.35
**SYSU GY3-64**	Y3	SCM-*Thermus* 162 medium	Group 30	*Thermus caliditerrae*	YIM 77925(T)	99.35
**SYSU GY3-68**	Y3	SCM-*Thermus* 162 medium	Group 30	*Thermus caliditerrae*	YIM 77925(T)	99.35
**SYSU GY3-69**	Y3	SCM-*Thermus* 162 medium	Group 30	*Thermus caliditerrae*	YIM 77925(T)	99.35
**SYSU GY3-71**	Y3	SCM-*Thermus* 162 medium	Group 30	*Thermus caliditerrae*	YIM 77925(T)	99.35
**SYSU GY3-74**	Y3	SCM-*Thermus* 162 medium	Group 30	*Thermus caliditerrae*	YIM 77925(T)	99.35
**SYSU GY3-94**	Y3	SCM-*Thermus* 162 medium	Group 30	*Thermus caliditerrae*	YIM 77925(T)	99.35
**SYSU GY3-189**	Y3	*Thermus* 162 medium	Group 30	*Thermus caliditerrae*	YIM 77925(T)	99.35
**SYSU GY3-190**	Y3	*Thermus* 162 medium	Group 30	*Thermus caliditerrae*	YIM 77925(T)	99.35
**SYSU GY3-153**	Y3	SCM-R2A	Group 30	*Thermus caliditerrae*	YIM 77925(T)	99.35
**SYSU GY3-P5**	Y3	SCM-*Thermus* 162 medium	Group 11	*Thermaerobacter subterraneus*	DSM 13965(T)	99.35
**SYSU GY3-48**	Y3	R2A	Group 30	*Thermus caliditerrae*	YIM 77925(T)	99.36
**SYSU GY3-108**	Y3	*Thermus* 162 medium	Group 30	*Thermus caliditerrae*	YIM 77925(T)	99.36
**SYSU GY3-65**	Y3	SCM-*Thermus* 162 medium	Group 30	*Thermus caliditerrae*	YIM 77925(T)	99.36
**SYSU GY3-78**	Y3	*Thermus* 162 medium	Group 30	*Thermus caliditerrae*	YIM 77925(T)	99.37
**SYSU GY3-93**	Y3	SCM-*Thermus* 162 medium	Group 30	*Thermus caliditerrae*	YIM 77925(T)	99.37
**SYSU GY3-95**	Y3	SCM-*Thermus* 162 medium	Group 30	*Thermus caliditerrae*	YIM 77925(T)	99.37
**SYSU GY3-113**	Y3	SCM-R2A	Group 30	*Thermus caliditerrae*	YIM 77925(T)	99.42
**SYSU GY3-2**	Y3	SCM-R2A	Group 40	*Chloroflexus islandicus*	isl-2(T)	99.48
**SYSU GY3-34**	Y3	SCM-R2A	Group 40	*Chloroflexus islandicus*	isl-2(T)	99.48
**SYSU GY3-127**	Y3	SCM-R2A	Group 40	*Chloroflexus islandicus*	isl-2(T)	99.48

**SYSU GY3-104-2**	Y3	SCM-*Thermus* 162 medium	Group 16	*Caldilinea tarbellica*	D1-25-10-4(T)	99.49
**SYSU GY3-87**	Y3	*Thermus* 162 medium	Group 30	*Thermus caliditerrae*	YIM 77925(T)	99.6
**SYSU GY3-6**	Y3	SCM-R2A	Group 16	*Caldilinea tarbellica*	D1-25-10-4(T)	99.61
**SYSU GY3-103**	Y3	SCM-*Thermus* 162 medium	Group 17	*Anoxybacillus mongoliensis*	T4(T)	99.61
**SYSU GY3-99**	Y3	SCM-*Thermus* 162 medium	Group 30	*Thermus caliditerrae*	YIM 77925(T)	99.73
**SYSU GY3-11**	Y3	SCM-R2A	Group 16	*Caldilinea tarbellica*	D1-25-10-4(T)	99.74
**SYSU GY3-12**	Y3	SCM-R2A	Group 16	*Caldilinea tarbellica*	D1-25-10-4(T)	99.74
**SYSU GY3-181**	Y3	SCM-R2A	Group 16	*Caldilinea tarbellica*	D1-25-10-4(T)	99.74
**SYSU GY3-21**	Y3	SCM-R2A	Group 29	*Thermus tengchongensis*	YIM 77924(T)	99.74
**SYSU GY3-104-3**	Y3	SCM-*Thermus* 162 medium	Group 17	*Anoxybacillus mongoliensis*	T4(T)	99.74
**SYSU GY3-105**	Y3	SCM-*Thermus* 162 medium	Group 17	*Anoxybacillus mongoliensis*	T4(T)	99.74
**SYSU GY3-124**	Y3	SCM-R2A	Group 29	*Thermus tengchongensis*	YIM 77924(T)	99.87
**SYSU GY3-128**	Y3	SCM-R2A	Group 29	*Thermus tengchongensis*	YIM 77924(T)	99.87
**SYSU GY3-79**	Y3	*Thermus* 162 medium	Group 31	*Tepidicella xavieri*	TU-16(T)	99.87
**SYSU GY3-25**	Y3	SCM-R2A	Group 29	*Thermus tengchongensis*	YIM 77924(T)	100

continued Supplementary Table 4：

**Strain ID**	**Top-hit taxonomy**	**Relative abundance in community（%）**
**SYSU GT96-39**	Bacteria;Bacteroidetes;Sphingobacteriia;CP012155;CP012155;FJ529921	ND
**SYSU GT96-1**	Bacteria;Bacteroidetes;Flavobacteria;Flavobacteriales;Schleiferiaceae;Schleiferia	0.776593981
**SYSU GT96-37**	Bacteria;Bacteroidetes;Flavobacteria;Flavobacteriales;Schleiferiaceae;Schleiferia	0.776593981
**SYSU GT96-40-1**	Bacteria;Bacteroidetes;Cytophagia;Cytophagales;Cytophagaceae;AF445665	ND
**SYSU GT96-46**	Bacteria;Proteobacteria;Alphaproteobacteria;Sphingomonadales;Sphingomonadaceae;Sandaracinobacter	0.773976248
**SYSU GT96-18**	Bacteria;Proteobacteria;Alphaproteobacteria;Sphingomonadales;Sphingomonadaceae;Sandaracinobacter	0.773976248
**SYSU GT96-61**	Bacteria;Proteobacteria;Alphaproteobacteria;Sphingomonadales;Sphingomonadaceae;Sandaracinobacter	0.773976248
**SYSU GT96-4**	Bacteria;Proteobacteria;Alphaproteobacteria;Sphingomonadales;Sphingomonadaceae;Sandaracinobacter	0.773976248
**SYSU GT96-72**	Bacteria;Proteobacteria;Alphaproteobacteria;Sphingomonadales;Sphingomonadaceae;Sandaracinobacter	0.773976248

**SYSU GT96-53**	Bacteria;Proteobacteria;Alphaproteobacteria;Sphingomonadales;Sphingomonadaceae;Sandaracinobacter	0.773976248
**SYSU GT96-54**	Bacteria;Proteobacteria;Alphaproteobacteria;Sphingomonadales;Sphingomonadaceae;Sandaracinobacter	0.773976248
**SYSU GT96-34**	Bacteria;Proteobacteria;Alphaproteobacteria;Sphingomonadales;Sphingomonadaceae;Sandaracinobacter	0.773976248
**SYSU GT96-47**	Bacteria;Proteobacteria;Alphaproteobacteria;Sphingomonadales;Sphingomonadaceae;Sandaracinobacter	0.773976248
**SYSU GT96-57**	Bacteria;Proteobacteria;Alphaproteobacteria;Sphingomonadales;Sphingomonadaceae;Sandaracinobacter	0.773976248
**SYSU GT96-56**	Bacteria;Proteobacteria;Alphaproteobacteria;Sphingomonadales;Sphingomonadaceae;Sandaracinobacter	0.773976248
**SYSU GT96-71**	Bacteria;Proteobacteria;Alphaproteobacteria;Sphingomonadales;Sphingomonadaceae;Sandaracinobacter	0.773976248
**SYSU GT96-34-1**	Bacteria;Proteobacteria;Alphaproteobacteria;Rhodospirillales;Acetobacteraceae;Roseomonas	ND
**SYSU GT96-19**	Bacteria;Chloroflexi;Chloroflexia;Chloroflexales;Chloroflexaceae;Chloroflexus	28.16505676
**SYSU GT96-45**	Bacteria;Chloroflexi;Chloroflexia;Chloroflexales;Chloroflexaceae;Chloroflexus	28.16505676
**SYSU GT96-3**	Bacteria;Chloroflexi;Chloroflexia;Chloroflexales;Chloroflexaceae;Chloroflexus	28.16505676
**SYSU GT96-40**	Bacteria;Chloroflexi;Chloroflexia;Chloroflexales;Chloroflexaceae;Chloroflexus	28.16505676
**SYSU GT96-52**	Bacteria;Chloroflexi;Chloroflexia;Chloroflexales;Chloroflexaceae;Chloroflexus	28.16505676
**SYSU GT96-20**	Bacteria;Chloroflexi;Chloroflexia;Chloroflexales;Chloroflexaceae;Chloroflexus	28.16505676
**SYSU GT96-17**	Bacteria;Proteobacteria;Betaproteobacteria;Burkholderiales;Comamonadaceae;AM777983	ND
**SYSU GT96-60**	Bacteria;Proteobacteria;Alphaproteobacteria;Rhodospirillales;Acetobacteraceae;Elioraea	1.166636126
**SYSU GT96-24**	Bacteria;Proteobacteria;Alphaproteobacteria;Rhodospirillales;Acetobacteraceae;Elioraea	1.166636126
**SYSU GT96-14**	Bacteria;Proteobacteria;Alphaproteobacteria;Rhodospirillales;Acetobacteraceae;Roseomonas	0.027049903
**SYSU GT96-26**	Bacteria;Firmicutes;Bacilli;Bacillales;Bacillaceae;Bacillus	ND
**SYSU GT96-12**	Bacteria;Firmicutes;Bacilli;Bacillales;Bacillaceae;Bacillus	ND
**SYSU GY1-61**	Bacteria;Proteobacteria;Alphaproteobacteria;Rhodospirillales;Alysiosphaera;FM242433	ND
**SYSU GY1-62**	Bacteria;Proteobacteria;Alphaproteobacteria;Rhodospirillales;Alysiosphaera;FM242433	ND
**SYSU GY1-30**	Bacteria;Bacteroidetes;Flavobacteria;Flavobacteriales;Schleiferiaceae;Schleiferia	0.776593981
**SYSU GY1-233**	Bacteria;Proteobacteria;Alphaproteobacteria;Rhodospirillales;Acetobacteraceae;Craurococcus	ND
**SYSU GY1-259**	Bacteria;Proteobacteria;Alphaproteobacteria;Rhodospirillales;Acetobacteraceae;Craurococcus	ND
**SYSU GY1-P12**	Bacteria;Actinobacteria;Acidimicrobiia;Acidimicrobiales;Iamiaceae;EF516779	ND
**SYSU GY1-29**	Bacteria;Chloroflexi;Chloroflexia;Chloroflexales;Chloroflexaceae;Chloroflexus	28.16505676
**SYSU GY1-23**	Bacteria;Proteobacteria;Alphaproteobacteria;Rhodospirillales;Acetobacteraceae;Elioraea	ND

**SYSU GY1-75**	Bacteria;Proteobacteria;Alphaproteobacteria;Sphingomonadales;Sphingomonadaceae;Sandaracinobacter	0.773976248
**SYSU GY1-100**	Bacteria;Actinobacteria;Acidimicrobiia;Acidimicrobiales;Iamiaceae;EF632905	ND
**SYSU GY1-247**	Bacteria;Deinococcus-Thermus;Deinococci;Thermales;Thermaceae;Meiothermus	0.596843015
**SYSU GY1-96**	Bacteria;Actinobacteria;Acidimicrobiia;Acidimicrobiales;Iamiaceae;EF632905	ND
**SYSU GY1-59**	Bacteria;Deinococcus-Thermus;Deinococci;Thermales;Thermaceae;Thermus	8.070469359
**SYSU GY1-65**	Bacteria;Chloroflexi;Chloroflexia;Chloroflexales;Roseiflexaceae;Roseiflexus	7.817421882
**SYSU GY1-4**	Bacteria;Proteobacteria;Betaproteobacteria;Burkholderiales;Comamonadaceae;Tepidimonas	0.724239331
**SYSU GY1-28**	Bacteria;Proteobacteria;Betaproteobacteria;Burkholderiales;Comamonadaceae;Tepidimonas	0.724239331
**SYSU GY1-58**	Bacteria;Proteobacteria;Betaproteobacteria;Burkholderiales;Comamonadaceae;Tepidimonas	0.724239331
**SYSU GY1-64**	Bacteria;Proteobacteria;Betaproteobacteria;Burkholderiales;Comamonadaceae;Tepidimonas	0.724239331
**SYSU GY1-60**	Bacteria;Proteobacteria;Betaproteobacteria;Burkholderiales;Comamonadaceae;Tepidimonas	0.724239331
**SYSU GY1-63**	Bacteria;Proteobacteria;Betaproteobacteria;Burkholderiales;Comamonadaceae;Tepidimonas	0.724239331
**SYSU GY1-278**	Bacteria;Proteobacteria;Gammaproteobacteria;Xanthomonadales;Xanthomonadaceae;AF407725	ND
**SYSU GY1-272**	Bacteria;Proteobacteria;Gammaproteobacteria;Xanthomonadales;Xanthomonadaceae;AF407725	ND
**SYSU GY1-174**	Bacteria;Deinococcus-Thermus;Deinococci;Thermales;Thermaceae;Meiothermus	0.596843015
**SYSU GY1-105**	Bacteria;Proteobacteria;Alphaproteobacteria;Rhodospirillales;Acetobacteraceae;Roseomonas	ND
**SYSU GY1-82**	Bacteria;Proteobacteria;Alphaproteobacteria;Rhodospirillales;Acetobacteraceae;Roseomonas	ND
**SYSU GY1-241**	Bacteria;Proteobacteria;Alphaproteobacteria;Rhodospirillales;Acetobacteraceae;Roseomonas	ND
**SYSU GY1-224**	Bacteria;Proteobacteria;Alphaproteobacteria;Rhodospirillales;Acetobacteraceae;Roseomonas	ND
**SYSU GY1-66**	Bacteria;Proteobacteria;Betaproteobacteria;Burkholderiales;Comamonadaceae;Tepidimonas	0.724239331
**SYSU GY1-56**	Bacteria;Proteobacteria;Betaproteobacteria;Burkholderiales;Comamonadaceae;Tepidimonas	0.724239331
**SYSU GY1-67**	Bacteria;Proteobacteria;Betaproteobacteria;Burkholderiales;Comamonadaceae;Tepidimonas	0.724239331
**SYSU GY1-57**	Bacteria;Proteobacteria;Betaproteobacteria;Burkholderiales;Comamonadaceae;Tepidimonas	0.724239331
**SYSU GY1-239**	Bacteria;Acidobacteria;Solibacteres;Solibacterales;Bryobacteraceae;Paludibaculum	ND
**SYSU GY1-242**	Bacteria;Acidobacteria;Solibacteres;Solibacterales;Bryobacteraceae;Paludibaculum	ND
**SYSU GY1-67P**	Bacteria;Proteobacteria;Betaproteobacteria;Burkholderiales;Comamonadaceae;Tepidimonas	0.724239331
**SYSU GY1-38P**	Bacteria;Deinococcus-Thermus;Deinococci;Thermales;Thermaceae;Thermus	8.070469359
**SYSU GY1-99**	Bacteria;Deinococcus-Thermus;Deinococci;Thermales;Thermaceae;Thermus	8.070469359

**SYSU GY1-170**	Bacteria;Deinococcus-Thermus;Deinococci;Thermales;Thermaceae;Meiothermus	0.596843015
**SYSU GY1-122**	Bacteria;Deinococcus-Thermus;Deinococci;Thermales;Thermaceae;Thermus	8.070469359
**SYSU GY1-284**	Bacteria;Deinococcus-Thermus;Deinococci;Thermales;Thermaceae;Meiothermus	0.596843015
**SYSU GY1-252**	Bacteria;Deinococcus-Thermus;Deinococci;Thermales;Thermaceae;Thermus	8.070469359
**SYSU GY1-283**	Bacteria;Deinococcus-Thermus;Deinococci;Thermales;Thermaceae;Thermus	8.070469359
**SYSU GY1-222**	Bacteria;Deinococcus-Thermus;Deinococci;Thermales;Thermaceae;Meiothermus	0.596843015
**SYSU GY1-182**	Bacteria;Deinococcus-Thermus;Deinococci;Thermales;Thermaceae;Meiothermus	0.596843015
**SYSU GY1-253**	Bacteria;Deinococcus-Thermus;Deinococci;Thermales;Thermaceae;Meiothermus	0.596843015
**SYSU GY1-254**	Bacteria;Deinococcus-Thermus;Deinococci;Thermales;Thermaceae;Meiothermus	0.596843015
**SYSU GY1-277**	Bacteria;Deinococcus-Thermus;Deinococci;Thermales;Thermaceae;Thermus	8.070469359
**SYSU GY1-257**	Bacteria;Deinococcus-Thermus;Deinococci;Thermales;Thermaceae;Meiothermus	0.596843015
**SYSU GY1-258**	Bacteria;Deinococcus-Thermus;Deinococci;Thermales;Thermaceae;Meiothermus	0.596843015
**SYSU GY1-164**	Bacteria;Deinococcus-Thermus;Deinococci;Thermales;Thermaceae;Meiothermus	0.596843015
**SYSU GY1-5**	Bacteria;Chloroflexi;Chloroflexia;Chloroflexales;Chloroflexaceae;Chloroflexus	28.16505676
**SYSU GY1-P29**	Bacteria;Actinobacteria;Thermoleophilia;Thermoleophilales;Thermoleophilaceae;Thermoleophilum	0.015706395
**SYSU GY1-83**	Bacteria;Proteobacteria;Betaproteobacteria;Burkholderiales;Comamonadaceae;AM777983	ND
**SYSU GY1-86**	Bacteria;Proteobacteria;Betaproteobacteria;Burkholderiales;Comamonadaceae;AM777983	ND
**SYSU GY1-106**	Bacteria;Proteobacteria;Betaproteobacteria;Burkholderiales;Comamonadaceae;AM777983	ND
**SYSU GY1-236**	Bacteria;Proteobacteria;Betaproteobacteria;Burkholderiales;Comamonadaceae;AM777983	ND
**SYSU GY1-141**	Bacteria;Proteobacteria;Betaproteobacteria;Burkholderiales;Comamonadaceae;AM777983	ND
**SYSU GY1-87**	Bacteria;Proteobacteria;Betaproteobacteria;Burkholderiales;Comamonadaceae;AM777983	ND
**SYSU GY1-149**	Bacteria;Proteobacteria;Betaproteobacteria;Burkholderiales;Comamonadaceae;AM777983	ND
**SYSU GY1-1**	Bacteria;Proteobacteria;Betaproteobacteria;Burkholderiales;Comamonadaceae;AM777983	ND
**SYSU GY1-251**	Bacteria;Deinococcus-Thermus;Deinococci;Thermales;Thermaceae;Thermus	8.070469359
**SYSU GY1-184**	Bacteria;Chloroflexi;Chloroflexia;Chloroflexales;Chloroflexaceae;Chloroflexus	28.16505676
**SYSU GY1-21**	Bacteria;Chloroflexi;Chloroflexia;Chloroflexales;Chloroflexaceae;Chloroflexus	28.16505676
**SYSU GY1-140**	Bacteria;Chloroflexi;Chloroflexia;Chloroflexales;Chloroflexaceae;Chloroflexus	28.16505676
**SYSU GY1-159**	Bacteria;Chloroflexi;Chloroflexia;Chloroflexales;Chloroflexaceae;Chloroflexus	28.16505676

**SYSU GY1-212**	Bacteria;Chloroflexi;Chloroflexia;Chloroflexales;Chloroflexaceae;Chloroflexus	28.16505676
**SYSU GY1-183**	Bacteria;Chloroflexi;Chloroflexia;Chloroflexales;Chloroflexaceae;Chloroflexus	28.16505676
**SYSU GY1-77**	Bacteria;Chloroflexi;Chloroflexia;Chloroflexales;Chloroflexaceae;Chloroflexus	28.16505676
**SYSU GY1-160**	Bacteria;Chloroflexi;Chloroflexia;Chloroflexales;Chloroflexaceae;Chloroflexus	28.16505676
**SYSU GY1-158**	Bacteria;Chloroflexi;Chloroflexia;Chloroflexales;Chloroflexaceae;Chloroflexus	28.16505676
**SYSU GY1-195**	Bacteria;Chloroflexi;Chloroflexia;Chloroflexales;Chloroflexaceae;Chloroflexus	28.16505676
**SYSU GY1-272-2**	Bacteria;Chloroflexi;Chloroflexia;Chloroflexales;Chloroflexaceae;Chloroflexus	28.16505676
**SYSU GY1-71**	Bacteria;Chloroflexi;EU335161;AB186887;AB186887;AB186887	ND
**SYSU GY1-179**	Bacteria;Proteobacteria;Gammaproteobacteria;Thiotrichales;Francisellaceae;Francisella	ND
**SYSU GY1-103**	Bacteria;Chloroflexi;EU335161;AB186887;AB186887;AB186887	ND
**SYSU GY1-84**	Bacteria;Chloroflexi;EU335161;AB186887;AB186887;AB186887	ND
**SYSU GY1-11**	Bacteria;Chloroflexi;Chloroflexia;Chloroflexales;Chloroflexaceae;Chloroflexus	28.16505676
**SYSU GY1-12**	Bacteria;Chloroflexi;Chloroflexia;Chloroflexales;Chloroflexaceae;Chloroflexus	28.16505676
**SYSU GY1-121**	Bacteria;Chloroflexi;Chloroflexia;Chloroflexales;Chloroflexaceae;Chloroflexus	28.16505676
**SYSU GY1-146**	Bacteria;Chloroflexi;Chloroflexia;Chloroflexales;Chloroflexaceae;Chloroflexus	28.16505676
**SYSU GY1-22**	Bacteria;Chloroflexi;EU335161;AB186887;AB186887;AB186887	ND
**SYSU GY1-68**	Bacteria;Chloroflexi;EU335161;AB186887;AB186887;AB186887	ND
**SYSU GY1-69**	Bacteria;Chloroflexi;EU335161;AB186887;AB186887;AB186887	ND
**SYSU GY1-228**	Bacteria;Deinococcus-Thermus;Deinococci;Thermales;Thermaceae;Thermus	8.070469359
**SYSU GY1-55**	Bacteria;Deinococcus-Thermus;Deinococci;Thermales;Thermaceae;Thermus	8.070469359
**SYSU GY1-57-2**	Bacteria;Deinococcus-Thermus;Deinococci;Thermales;Thermaceae;Thermus	8.070469359
**SYSU GY1-19**	Bacteria;Chloroflexi;EU335161;AB186887;AB186887;AB186887	ND
**SYSU GY1-156**	Bacteria;Chloroflexi;Chloroflexia;Chloroflexales;Chloroflexaceae;Chloroflexus	28.16505676
**SYSU GY1-157**	Bacteria;Chloroflexi;Chloroflexia;Chloroflexales;Chloroflexaceae;Chloroflexus	28.16505676
**SYSU GY1-109**	Bacteria;Chloroflexi;EU335161;AB186887;AB186887;AB186887	ND
**SYSU GY1-80**	Bacteria;Proteobacteria;Alphaproteobacteria;Rhodospirillales;Alysiosphaera;AP011749	ND
**SYSU GY1-89**	Bacteria;Chloroflexi;Chloroflexia;Chloroflexales;Chloroflexaceae;Chloroflexus	28.16505676
**SYSU GY1-178**	Bacteria;Chloroflexi;Chloroflexia;Chloroflexales;Chloroflexaceae;Chloroflexus	28.16505676

**SYSU GY1-74**	Bacteria;Proteobacteria;Alphaproteobacteria;Rhodospirillales;Acetobacteraceae;Elioraea	1.166636126
**SYSU GYI-238-2**	Bacteria;Proteobacteria;Betaproteobacteria;Hydrogenophilales;Thiobacter;Thiobacter	0.116925386
**SYSU GY1-173**	Bacteria;Deinococcus-Thermus;Deinococci;Thermales;Thermaceae;Thermus	8.070469359
**SYSU GY1-136**	Bacteria;Firmicutes;Bacilli;Bacillales;Bacillaceae;Bacillus	0.066315891
**SYSU GY1-139**	Bacteria;Firmicutes;Bacilli;Bacillales;Bacillaceae;Bacillus	0.066315891
**SYSU GY1-176**	Bacteria;Firmicutes;Bacilli;Bacillales;Bacillaceae;Bacillus	0.066315891
**SYSU GY1-273**	Bacteria;Firmicutes;Bacilli;Bacillales;Bacillaceae;Bacillus	0.066315891
**SYSU GY1-35-1**	Bacteria;Proteobacteria;Betaproteobacteria;Burkholderiales;Comamonadaceae;Caldimonas	ND
**SYSU GY1-36**	Bacteria;Proteobacteria;Betaproteobacteria;Burkholderiales;Comamonadaceae;Caldimonas	ND
**SYSU GY1-37**	Bacteria;Proteobacteria;Betaproteobacteria;Burkholderiales;Comamonadaceae;Caldimonas	ND
**SYSU GY1-211**	Bacteria;Proteobacteria;Betaproteobacteria;Burkholderiales;Comamonadaceae;Tepidimonas	0.724239331
**SYSU GY1-73**	Bacteria;Proteobacteria;Betaproteobacteria;Burkholderiales;Comamonadaceae;Tepidimonas	0.724239331
**SYSU GY1-226**	Bacteria;Deinococcus-Thermus;Deinococci;Thermales;Thermaceae;Thermus	8.070469359
**SYSU GY1-243**	Bacteria;Deinococcus-Thermus;Deinococci;Thermales;Thermaceae;Thermus	8.070469359
**SYSU GY1-128**	Bacteria;Deinococcus-Thermus;Deinococci;Thermales;Thermaceae;Thermus	8.070469359
**SYSU GY1-175**	Bacteria;Deinococcus-Thermus;Deinococci;Thermales;Thermaceae;Thermus	8.070469359
**SYSU GY1-181**	Bacteria;Deinococcus-Thermus;Deinococci;Thermales;Thermaceae;Thermus	8.070469359
**SYSU GY1-78**	Bacteria;Deinococcus-Thermus;Deinococci;Thermales;Thermaceae;Thermus	8.070469359
**SYSU GY1-90**	Bacteria;Deinococcus-Thermus;Deinococci;Thermales;Thermaceae;Thermus	8.070469359
**SYSU GY1-91**	Bacteria;Deinococcus-Thermus;Deinococci;Thermales;Thermaceae;Thermus	8.070469359
**SYSU GY1-54**	Bacteria;Deinococcus-Thermus;Deinococci;Thermales;Thermaceae;Thermus	8.070469359
**SYSU GY1-276**	Bacteria;Deinococcus-Thermus;Deinococci;Thermales;Thermaceae;Thermus	8.070469359
**SYSU GY1-232**	Bacteria;Proteobacteria;Betaproteobacteria;Hydrogenophilales;Thiobacter;Thiobacter	0.116925386
**SYSU GY1-234**	Bacteria;Proteobacteria;Betaproteobacteria;Hydrogenophilales;Thiobacter;Thiobacter	0.116925386
**SYSU GY2-132**	Bacteria;Proteobacteria;Alphaproteobacteria;Rhodospirillales;Acetobacteraceae;Craurococcus	ND
**SYSU GY2-90**	Bacteria;Chloroflexi;Chloroflexia;Chloroflexales;Roseiflexaceae;Roseiflexus	7.817421882
**SYSU GY2-8**	Bacteria;Chloroflexi;Chloroflexia;Chloroflexales;Roseiflexaceae;Roseiflexus	7.817421882
**SYSU GY2-10**	Bacteria;Chloroflexi;Chloroflexia;Chloroflexales;Roseiflexaceae;Roseiflexus	7.817421882

**SYSU GY2-99**	Bacteria;Chloroflexi;Chloroflexia;Chloroflexales;Roseiflexaceae;Roseiflexus	7.817421882
**SYSU GY2-6**	Bacteria;Chloroflexi;Chloroflexia;Chloroflexales;Roseiflexaceae;Roseiflexus	7.817421882
**SYSU GY2-106**	Bacteria;Deinococcus-Thermus;Deinococci;Thermales;Thermaceae;Thermus	ND
**SYSU GY2-123**	Bacteria;Deinococcus-Thermus;Deinococci;Thermales;Thermaceae;Thermus	ND
**SYSU GY2-124**	Bacteria;Deinococcus-Thermus;Deinococci;Thermales;Thermaceae;Thermus	ND
**SYSU GY2-136**	Bacteria;Deinococcus-Thermus;Deinococci;Thermales;Thermaceae;Thermus	ND
**SYSU GY2-110**	Bacteria;Deinococcus-Thermus;Deinococci;Thermales;Thermaceae;Thermus	ND
**SYSU GY2-121**	Bacteria;Deinococcus-Thermus;Deinococci;Thermales;Thermaceae;Thermus	ND
**SYSU GY2-86**	Bacteria;Deinococcus-Thermus;Deinococci;Thermales;Thermaceae;Thermus	ND
**SYSU GY2-115**	Bacteria;Deinococcus-Thermus;Deinococci;Thermales;Thermaceae;Thermus	ND
**SYSU GY2-32**	Bacteria;Proteobacteria;Betaproteobacteria;Burkholderiales;Comamonadaceae;Tepidimonas	0.724239331
**SYSU GY2-128**	Bacteria;Proteobacteria;Gammaproteobacteria;Xanthomonadales;Xanthomonadaceae;AF407725	ND
**SYSU GY2-12**	Bacteria;Deinococcus-Thermus;Deinococci;Thermales;Thermaceae;Meiothermus	0.596843015
**SYSU GY2-45**	Bacteria;Proteobacteria;Betaproteobacteria;Burkholderiales;Comamonadaceae;Tepidimonas	0.724239331
**SYSU GY2-68**	Bacteria;Deinococcus-Thermus;Deinococci;Thermales;Thermaceae;Thermus	8.070469359
**SYSU GY2-54**	Bacteria;Chloroflexi;EU335161;AB186887;AB186887;AB186887	ND
**SYSU GY2-42**	Bacteria;Deinococcus-Thermus;Deinococci;Thermales;Thermaceae;Thermus	8.070469359
**SYSU GY2-29？**	Bacteria;Deinococcus-Thermus;Deinococci;Thermales;Thermaceae;Thermus	8.070469359
**SYSU GY2-104**	Bacteria;Chloroflexi;Chloroflexia;Chloroflexales;Chloroflexaceae;Chloroflexus	28.16505676
**SYSU GY2-88**	Bacteria;Chloroflexi;Chloroflexia;Chloroflexales;Chloroflexaceae;Chloroflexus	28.16505676
**SYSU GY2-135**	Bacteria;Chloroflexi;Chloroflexia;Chloroflexales;Chloroflexaceae;Chloroflexus	28.16505676
**SYSU GY2-39**	Bacteria;Deinococcus-Thermus;Deinococci;Thermales;Thermaceae;Thermus	8.070469359
**SYSU GY2-83**	Bacteria;Chloroflexi;Chloroflexia;Chloroflexales;Chloroflexaceae;Chloroflexus	28.16505676
**SYSU GY2-66**	Bacteria;Deinococcus-Thermus;Deinococci;Thermales;Thermaceae;Thermus	8.070469359
**SYSU GY2-76**	Bacteria;Deinococcus-Thermus;Deinococci;Thermales;Thermaceae;Thermus	8.070469359
**SYSU GY2-3**	Bacteria;Deinococcus-Thermus;Deinococci;Thermales;Thermaceae;Thermus	8.070469359
**SYSU GY2-67**	Bacteria;Deinococcus-Thermus;Deinococci;Thermales;Thermaceae;Thermus	8.070469359
**SYSU GY2-27**	Bacteria;Deinococcus-Thermus;Deinococci;Thermales;Thermaceae;Thermus	8.070469359

**SYSU GY2-33**	Bacteria;Deinococcus-Thermus;Deinococci;Thermales;Thermaceae;Thermus	8.070469359
**SYSU GY2-44**	Bacteria;Deinococcus-Thermus;Deinococci;Thermales;Thermaceae;Thermus	8.070469359
**SYSU GY2-9**	Bacteria;Deinococcus-Thermus;Deinococci;Thermales;Thermaceae;Thermus	8.070469359
**SYSU GY2-93**	Bacteria;Deinococcus-Thermus;Deinococci;Thermales;Thermaceae;Thermus	8.070469359
**SYSU GY2-120**	Bacteria;Deinococcus-Thermus;Deinococci;Thermales;Thermaceae;Thermus	8.070469359
**SYSU GY2-129**	Bacteria;Deinococcus-Thermus;Deinococci;Thermales;Thermaceae;Thermus	8.070469359
**SYSU GY2-141**	Bacteria;Deinococcus-Thermus;Deinococci;Thermales;Thermaceae;Thermus	8.070469359
**SYSU GY2-20**	Bacteria;Deinococcus-Thermus;Deinococci;Thermales;Thermaceae;Thermus	8.070469359
**SYSU GY2-31**	Bacteria;Deinococcus-Thermus;Deinococci;Thermales;Thermaceae;Thermus	8.070469359
**SYSU GY2-35**	Bacteria;Deinococcus-Thermus;Deinococci;Thermales;Thermaceae;Thermus	8.070469359
**SYSU GY2-40**	Bacteria;Deinococcus-Thermus;Deinococci;Thermales;Thermaceae;Thermus	8.070469359
**SYSU GY2-46**	Bacteria;Deinococcus-Thermus;Deinococci;Thermales;Thermaceae;Thermus	8.070469359
**SYSU GY2-60**	Bacteria;Deinococcus-Thermus;Deinococci;Thermales;Thermaceae;Thermus	8.070469359
**SYSU GY2-62**	Bacteria;Deinococcus-Thermus;Deinococci;Thermales;Thermaceae;Thermus	8.070469359
**SYSU GY2-69**	Bacteria;Deinococcus-Thermus;Deinococci;Thermales;Thermaceae;Thermus	8.070469359
**SYSU GY2-98**	Bacteria;Firmicutes;Bacilli;Bacillales;Bacillaceae;Bacillus	0.066315891
**SYSU GY2-155**	Bacteria;Firmicutes;Bacilli;Bacillales;Bacillaceae;Bacillus	0.066315891
**SYSU GY2-148**	Bacteria;Firmicutes;Bacilli;Bacillales;Bacillaceae;Bacillus	0.066315891
**SYSU GY2-14**	Bacteria;Deinococcus-Thermus;Deinococci;Thermales;Thermaceae;Thermus	8.070469359
**SYSU GY2-75**	Bacteria;Deinococcus-Thermus;Deinococci;Thermales;Thermaceae;Thermus	8.070469359
**SYSU GY2-48**	Bacteria;Deinococcus-Thermus;Deinococci;Thermales;Thermaceae;Thermus	8.070469359
**SYSU GY2-89**	Bacteria;Deinococcus-Thermus;Deinococci;Thermales;Thermaceae;Thermus	8.070469359
**SYSU GY2-97**	Bacteria;Deinococcus-Thermus;Deinococci;Thermales;Thermaceae;Thermus	8.070469359
**SYSU GY2-105**	Bacteria;Deinococcus-Thermus;Deinococci;Thermales;Thermaceae;Thermus	8.070469359
**SYSU GY2-140**	Bacteria;Deinococcus-Thermus;Deinococci;Thermales;Thermaceae;Thermus	8.070469359
**SYSU GY2-34**	Bacteria;Deinococcus-Thermus;Deinococci;Thermales;Thermaceae;Thermus	8.070469359
**SYSU GY2-47**	Bacteria;Deinococcus-Thermus;Deinococci;Thermales;Thermaceae;Thermus	8.070469359
**SYSU GY2-58**	Bacteria;Deinococcus-Thermus;Deinococci;Thermales;Thermaceae;Thermus	8.070469359

**SYSU GY3-13**	Bacteria;Proteobacteria;Alphaproteobacteria;Rhodospirillales;Acetobacteraceae;Elioraea	ND
**SYSU GY3-101**	Bacteria;Chloroflexi;Chloroflexia;Chloroflexales;Chloroflexaceae;Chloroflexus	28.16505676
**SYSU GY3-23**	Bacteria;Bacteroidetes;Flavobacteria;Flavobacteriales;Schleiferiaceae;Schleiferia	0.776593981
**SYSU GY3-42**	Bacteria;Proteobacteria;Alphaproteobacteria;Sphingomonadales;Sphingomonadaceae;Sandaracinobacter	0.773976248
**SYSU GY3-136**	Bacteria;Proteobacteria;Alphaproteobacteria;Sphingomonadales;Sphingomonadaceae;Sandaracinobacter	0.773976248
**SYSU GY3-35**	Bacteria;Proteobacteria;Alphaproteobacteria;Sphingomonadales;Sphingomonadaceae;Sandaracinobacter	0.773976248
**SYSU GY3-37**	Bacteria;Proteobacteria;Alphaproteobacteria;Sphingomonadales;Sphingomonadaceae;Sandaracinobacter	0.773976248
**SYSU GY-37**	Bacteria;Proteobacteria;Alphaproteobacteria;Sphingomonadales;Sphingomonadaceae;Sandaracinobacter	0.773976248
**SYSU GY3-P8**	Bacteria;Chloroflexi;Chloroflexia;Chloroflexales;Roseiflexaceae;Roseiflexus	7.817421882
**SYSU GY3-138**	Bacteria;Chloroflexi;Chloroflexia;Chloroflexales;Roseiflexaceae;Roseiflexus	7.817421882
**SYSU GY3-144**	Bacteria;Chloroflexi;Chloroflexia;Chloroflexales;Roseiflexaceae;Roseiflexus	ND
**SYSU GY3-41**	Bacteria;Proteobacteria;Alphaproteobacteria;Rhodospirillales;Acetobacteraceae;Elioraea	1.166636126
**SYSU GY3-147**	Bacteria;Deinococcus-Thermus;Deinococci;Thermales;Thermaceae;Thermus	ND
**SYSU GY3-17**	Bacteria;Chloroflexi;Chloroflexia;Chloroflexales;Chloroflexaceae;Chloroflexus	28.16505676
**SYSU GY3-112**	Bacteria;Deinococcus-Thermus;Deinococci;Thermales;Thermaceae;Thermus	ND
**SYSU GY3-6-2**	Bacteria;Chloroflexi;Chloroflexia;Chloroflexales;Roseiflexaceae;Roseiflexus	7.817421882
**SYSU GY3-63**	Bacteria;Proteobacteria;Gammaproteobacteria;Xanthomonadales;Xanthomonadaceae;AF407725	ND
**SYSU GY3-141**	Bacteria;Proteobacteria;Gammaproteobacteria;Xanthomonadales;Xanthomonadaceae;AF407725	ND
**SYSU GY3-81**	Bacteria;Aquificae;Aquificae;Aquificales;Hydrogenothermaceae;Sulfurihydrogenibium	ND
**SYSU GY3-83**	Bacteria;Aquificae;Aquificae;Aquificales;Hydrogenothermaceae;Sulfurihydrogenibium	ND
**SYSU GY3-67**	Bacteria;Aquificae;Aquificae;Aquificales;Hydrogenothermaceae;Sulfurihydrogenibium	ND
**SYSU GY3-146**	Bacteria;Deinococcus-Thermus;Deinococci;Thermales;Thermaceae;Thermus	8.070469359
**SYSU GY3-201**	Bacteria;Deinococcus-Thermus;Deinococci;Thermales;Thermaceae;Thermus	8.070469359
**SYSU GY3-61**	Bacteria;Proteobacteria;Betaproteobacteria;Burkholderiales;Comamonadaceae;Tepidimonas	0.724239331
**SYSU GY3-77**	Bacteria;Acidobacteria;Solibacteres;Solibacterales;Bryobacteraceae;Paludibaculum	ND
**SYSU GY3-85**	Bacteria;Acidobacteria;Solibacteres;Solibacterales;Bryobacteraceae;Paludibaculum	ND
**SYSU GY3-193**	Bacteria;Deinococcus-Thermus;Deinococci;Thermales;Thermaceae;Thermus	8.070469359
**SYSU GY3-150**	Bacteria;Deinococcus-Thermus;Deinococci;Thermales;Thermaceae;Thermus	8.070469359

**SYSU GY3-89**	Bacteria;Proteobacteria;Alphaproteobacteria;Rhodospirillales;Acetobacteraceae;Elioraea	ND
**SYSU GY3-38**	Bacteria;Deinococcus-Thermus;Deinococci;Thermales;Thermaceae;Thermus	8.070469359
**SYSU GY3-121**	Bacteria;Deinococcus-Thermus;Deinococci;Thermales;Thermaceae;Thermus	8.070469359
**SYSU GY3-194**	Bacteria;Deinococcus-Thermus;Deinococci;Thermales;Thermaceae;Thermus	8.070469359
**SYSU GY3-33**	Bacteria;Deinococcus-Thermus;Deinococci;Thermales;Thermaceae;Thermus	8.070469359
**SYSU GY3-31**	Bacteria;Deinococcus-Thermus;Deinococci;Thermales;Thermaceae;Thermus	8.070469359
**SYSU GY3-36**	Bacteria;Deinococcus-Thermus;Deinococci;Thermales;Thermaceae;Thermus	8.070469359
**SYSU GY3-192**	Bacteria;Deinococcus-Thermus;Deinococci;Thermales;Thermaceae;Thermus	8.070469359
**SYSU GY3-96**	Bacteria;Deinococcus-Thermus;Deinococci;Thermales;Thermaceae;Thermus	8.070469359
**SYSU GY3-14**	Bacteria;Deinococcus-Thermus;Deinococci;Thermales;Thermaceae;Thermus	8.070469359
**SYSU GY3-44**	Bacteria;Chloroflexi;Chloroflexia;Chloroflexales;Chloroflexaceae;Chloroflexus	28.16505676
**SYSU GY3-188**	Bacteria;Deinococcus-Thermus;Deinococci;Thermales;Thermaceae;Thermus	8.070469359
**SYSU GY3-196**	Bacteria;Deinococcus-Thermus;Deinococci;Thermales;Thermaceae;Thermus	8.070469359
**SYSU GY3-197**	Bacteria;Deinococcus-Thermus;Deinococci;Thermales;Thermaceae;Thermus	8.070469359
**SYSU GY3-199**	Bacteria;Deinococcus-Thermus;Deinococci;Thermales;Thermaceae;Thermus	8.070469359
**SYSU GY3-19**	Bacteria;Chloroflexi;EU335161;AB186887;AB186887;AB186887	ND
**SYSU GY3-20**	Bacteria;Chloroflexi;EU335161;AB186887;AB186887;AB186887	ND
**SYSU GY3-116**	Bacteria;Deinococcus-Thermus;Deinococci;Thermales;Thermaceae;Thermus	8.070469359
**SYSU GY3-59**	Bacteria;Deinococcus-Thermus;Deinococci;Thermales;Thermaceae;Thermus	8.070469359
**SYSU GY3-191**	Bacteria;Deinococcus-Thermus;Deinococci;Thermales;Thermaceae;Thermus	8.070469359
**SYSU GY3-195**	Bacteria;Deinococcus-Thermus;Deinococci;Thermales;Thermaceae;Thermus	8.070469359
**SYSU GY3-102**	Bacteria;Deinococcus-Thermus;Deinococci;Thermales;Thermaceae;Thermus	8.070469359
**SYSU GY3-142**	Bacteria;Chloroflexi;EU335161;AB186887;AB186887;AB186887	ND
**SYSU GY3-15**	Bacteria;Deinococcus-Thermus;Deinococci;Thermales;Thermaceae;Thermus	8.070469359
**SYSU GY3-49**	Bacteria;Chloroflexi;EU335161;AB186887;AB186887;AB186887	ND
**SYSU GY3-52**	Bacteria;Chloroflexi;EU335161;AB186887;AB186887;AB186887	ND
**SYSU GY3-53**	Bacteria;Chloroflexi;EU335161;AB186887;AB186887;AB186887	ND
**SYSU GY3-24**	Bacteria;Chloroflexi;EU335161;AB186887;AB186887;AB186887	ND

**SYSU GY3-143**	Bacteria;Chloroflexi;EU335161;AB186887;AB186887;AB186887	ND
**SYSU GY3-97**	Bacteria;Deinococcus-Thermus;Deinococci;Thermales;Thermaceae;Thermus	8.070469359
**SYSU GY3-39**	Bacteria;Deinococcus-Thermus;Deinococci;Thermales;Thermaceae;Thermus	8.070469359
**SYSU GY3-40**	Bacteria;Deinococcus-Thermus;Deinococci;Thermales;Thermaceae;Thermus	8.070469359
**SYSU GY3-41-2**	Bacteria;Deinococcus-Thermus;Deinococci;Thermales;Thermaceae;Thermus	8.070469359
**SYSU GY3-46**	Bacteria;Deinococcus-Thermus;Deinococci;Thermales;Thermaceae;Thermus	8.070469359
**SYSU GY3-47**	Bacteria;Deinococcus-Thermus;Deinococci;Thermales;Thermaceae;Thermus	8.070469359
**SYSU GY3-50**	Bacteria;Deinococcus-Thermus;Deinococci;Thermales;Thermaceae;Thermus	8.070469359
**SYSU GY3-51**	Bacteria;Deinococcus-Thermus;Deinococci;Thermales;Thermaceae;Thermus	8.070469359
**SYSU GY3-54**	Bacteria;Deinococcus-Thermus;Deinococci;Thermales;Thermaceae;Thermus	8.070469359
**SYSU GY3-55**	Bacteria;Deinococcus-Thermus;Deinococci;Thermales;Thermaceae;Thermus	8.070469359
**SYSU GY3-56**	Bacteria;Deinococcus-Thermus;Deinococci;Thermales;Thermaceae;Thermus	8.070469359
**SYSU GY3-86**	Bacteria;Deinococcus-Thermus;Deinococci;Thermales;Thermaceae;Thermus	8.070469359
**SYSU GY3-18**	Bacteria;Deinococcus-Thermus;Deinococci;Thermales;Thermaceae;Thermus	8.070469359
**SYSU GY3-30**	Bacteria;Deinococcus-Thermus;Deinococci;Thermales;Thermaceae;Thermus	8.070469359
**SYSU GY3-32**	Bacteria;Deinococcus-Thermus;Deinococci;Thermales;Thermaceae;Thermus	8.070469359
**SYSU GY3-118**	Bacteria;Deinococcus-Thermus;Deinococci;Thermales;Thermaceae;Thermus	8.070469359
**SYSU GY3-120**	Bacteria;Deinococcus-Thermus;Deinococci;Thermales;Thermaceae;Thermus	8.070469359
**SYSU GY3-126**	Bacteria;Deinococcus-Thermus;Deinococci;Thermales;Thermaceae;Thermus	8.070469359
**SYSU GY3-129**	Bacteria;Deinococcus-Thermus;Deinococci;Thermales;Thermaceae;Thermus	8.070469359
**SYSU GY3-57**	Bacteria;Deinococcus-Thermus;Deinococci;Thermales;Thermaceae;Thermus	8.070469359
**SYSU GY3-60**	Bacteria;Deinococcus-Thermus;Deinococci;Thermales;Thermaceae;Thermus	8.070469359
**SYSU GY3-62**	Bacteria;Deinococcus-Thermus;Deinococci;Thermales;Thermaceae;Thermus	8.070469359
**SYSU GY3-64**	Bacteria;Deinococcus-Thermus;Deinococci;Thermales;Thermaceae;Thermus	8.070469359
**SYSU GY3-68**	Bacteria;Deinococcus-Thermus;Deinococci;Thermales;Thermaceae;Thermus	8.070469359
**SYSU GY3-69**	Bacteria;Deinococcus-Thermus;Deinococci;Thermales;Thermaceae;Thermus	8.070469359
**SYSU GY3-71**	Bacteria;Deinococcus-Thermus;Deinococci;Thermales;Thermaceae;Thermus	8.070469359
**SYSU GY3-74**	Bacteria;Deinococcus-Thermus;Deinococci;Thermales;Thermaceae;Thermus	8.070469359

**SYSU GY3-94**	Bacteria;Deinococcus-Thermus;Deinococci;Thermales;Thermaceae;Thermus	8.070469359
**SYSU GY3-189**	Bacteria;Deinococcus-Thermus;Deinococci;Thermales;Thermaceae;Thermus	8.070469359
**SYSU GY3-190**	Bacteria;Deinococcus-Thermus;Deinococci;Thermales;Thermaceae;Thermus	8.070469359
**SYSU GY3-153**	Bacteria;Deinococcus-Thermus;Deinococci;Thermales;Thermaceae;Thermus	8.070469359
**SYSU GY3-P5**	Bacteria;Firmicutes;Clostridia;Thermoanaerobacterales;Thermaerobacter;Thermaerobacter	ND
**SYSU GY3-48**	Bacteria;Deinococcus-Thermus;Deinococci;Thermales;Thermaceae;Thermus	8.070469359
**SYSU GY3-108**	Bacteria;Deinococcus-Thermus;Deinococci;Thermales;Thermaceae;Thermus	8.070469359
**SYSU GY3-65**	Bacteria;Deinococcus-Thermus;Deinococci;Thermales;Thermaceae;Thermus	8.070469359
**SYSU GY3-78**	Bacteria;Deinococcus-Thermus;Deinococci;Thermales;Thermaceae;Thermus	8.070469359
**SYSU GY3-93**	Bacteria;Deinococcus-Thermus;Deinococci;Thermales;Thermaceae;Thermus	8.070469359
**SYSU GY3-95**	Bacteria;Deinococcus-Thermus;Deinococci;Thermales;Thermaceae;Thermus	8.070469359
**SYSU GY3-113**	Bacteria;Deinococcus-Thermus;Deinococci;Thermales;Thermaceae;Thermus	8.070469359
**SYSU GY3-2**	Bacteria;Chloroflexi;Chloroflexia;Chloroflexales;Chloroflexaceae;Chloroflexus	28.16505676
**SYSU GY3-34**	Bacteria;Chloroflexi;Chloroflexia;Chloroflexales;Chloroflexaceae;Chloroflexus	28.16505676
**SYSU GY3-127**	Bacteria;Chloroflexi;Chloroflexia;Chloroflexales;Chloroflexaceae;Chloroflexus	28.16505676
**SYSU GY3-104-2**	Bacteria;Chloroflexi;Caldilineae;Caldilineales;Caldilineaceae;Caldilinea	0.221634687
**SYSU GY3-87**	Bacteria;Deinococcus-Thermus;Deinococci;Thermales;Thermaceae;Thermus	8.070469359
**SYSU GY3-6**	Bacteria;Chloroflexi;Caldilineae;Caldilineales;Caldilineaceae;Caldilinea	0.221634687
**SYSU GY3-103**	Bacteria;Firmicutes;Bacilli;Bacillales;Bacillaceae;Anoxybacillus	0.157063951
**SYSU GY3-99**	Bacteria;Deinococcus-Thermus;Deinococci;Thermales;Thermaceae;Thermus	8.070469359
**SYSU GY3-11**	Bacteria;Chloroflexi;Caldilineae;Caldilineales;Caldilineaceae;Caldilinea	0.221634687
**SYSU GY3-12**	Bacteria;Chloroflexi;Caldilineae;Caldilineales;Caldilineaceae;Caldilinea	0.221634687
**SYSU GY3-181**	Bacteria;Chloroflexi;Caldilineae;Caldilineales;Caldilineaceae;Caldilinea	0.221634687
**SYSU GY3-21**	Bacteria;Deinococcus-Thermus;Deinococci;Thermales;Thermaceae;Thermus	8.070469359
**SYSU GY3-104-3**	Bacteria;Firmicutes;Bacilli;Bacillales;Bacillaceae;Anoxybacillus	0.157063951
**SYSU GY3-105**	Bacteria;Firmicutes;Bacilli;Bacillales;Bacillaceae;Anoxybacillus	0.157063951
**SYSU GY3-124**	Bacteria;Deinococcus-Thermus;Deinococci;Thermales;Thermaceae;Thermus	8.070469359
**SYSU GY3-128**	Bacteria;Deinococcus-Thermus;Deinococci;Thermales;Thermaceae;Thermus	8.070469359

**SYSU GY3-79**	Bacteria;Proteobacteria;Betaproteobacteria;Burkholderiales;Comamonadaceae;Tepidicella	0.073296511
**SYSU GY3-25**	Bacteria;Deinococcus-Thermus;Deinococci;Thermales;Thermaceae;Thermus	8.070469359

Note: ND, not detected in high throughput sequencing files.

Supplementary Table 5. List of metabolites detected in the spent culture medium (SCM).

Compound	ratio(W1/CK)	p-value	ratio(W2/CK)	p-value	ratio(W3/CK)	p-value	ratio(W4/CK)	p-value
	4 h		8 h		12 h		24 h	
Deoxycytidine	1.275581901	0.612927201	3.518705798	0.012640955	4.873195604	0.001526261	78.85635509	0.014671813
2'-deoxyadenosine	1.39508061	0.293103876	2.478473306	0.012245001	4.41492471	0.000352319	45.67379558	0.010206931
5'-deoxyadenosine	1.39508061	0.293103876	2.478473306	0.012245001	4.41492471	0.000352319	45.67379558	0.010206931
Deoxyadenosine	1.39508061	0.293103876	2.478473306	0.012245001	4.41492471	0.000352319	45.67379558	0.010206931
Muramic acid	1.39508061	0.293103876	2.478473306	0.012245001	4.41492471	0.000352319	45.67379558	0.010206931
Isopentenyladenine	2.857357979	0.078494146	17.355568	0.064426391	13.37271281	0.034081622	44.80751871	0.007641073
Cytosine	1.783774649	0.027654827	9.474045045	0.000736749	9.421251864	5.43979E-06	35.67788317	0.010838476
Indole acetaldehyde	1.133372456	0.511957036	13.80787892	3.40488E-05	6.883256698	1.29762E-06	13.63361952	0.008854468
N4-acetyl aminobutanal	1.188106517	0.425617279	2.847008357	0.012633844	2.002956712	0.010623608	9.130807179	0.002266932
Nicotinamide ribose	0.907915333	0.767301258	3.143833582	0.028667145	3.323933579	0.023705742	8.120664675	7.2039E-05
Pantothenic acid (VB5)	1.230365944	0.342226141	2.538930324	0.007733578	1.633019045	0.04041704	7.039690987	0.002513511
N-acetyl Glutamic acid	1.179977589	0.335302688	7.270944921	0.001032418	5.096344776	5.98678E-05	6.803259741	8.51656E-05
hydroxy Pyruvate	0.692766823	0.152222532	4.898986021	0.00074515	3.213084986	0.000433992	5.757009189	2.89393E-05
Malonate	0.692766823	0.152222532	4.898986021	0.00074515	3.213084986	0.000433992	5.757009189	2.89393E-05
L-Histidinol	1.66067784	0.060517162	4.047844853	0.003773859	2.602849744	0.004103524	0.247720245	0.023809787
N-acetyl Ornithine	1.137072726	0.511228807	2.351479006	0.014716974	1.8726147	0.010022495	0.186095547	0.046480523
2-amino Butyric acid	1.151958987	0.473777701	0.30095251	0.025563996	0.404755824	0.036356965	0.179596299	0.013519223
D-α-amino Butyric acid	1.151958987	0.473777701	0.30095251	0.025563996	0.404755824	0.036356965	0.179596299	0.013519223
L-α-amino Butyric acid (homoalanine)	1.151958987	0.473777701	0.30095251	0.025563996	0.404755824	0.036356965	0.179596299	0.013519223
γ-amino Butryic acid	1.151958987	0.473777701	0.30095251	0.025563996	0.404755824	0.036356965	0.179596299	0.013519223
Dimethylglycine	1.151958987	0.473777701	0.30095251	0.025563996	0.404755824	0.036356965	0.179596299	0.013519223
2-amino-2-methylpropanoate	1.151958987	0.473777701	0.298962119	0.025072049	0.404755824	0.036356965	0.179596299	0.013519223
3-aminoisobutanoate	1.151958987	0.473777701	0.298962119	0.025072049	0.404755824	0.036356965	0.179596299	0.013519223

4-aminobutanoate	1.151958987	0.473777701	0.298962119	0.025072049	0.404755824	0.036356965	0.179596299	0.013519223
α-keto Glutaric acid (Oxoglutaric acid)	0.997157209	0.988025222	0.080242842	0.02898861	0.159232688	0.03510424	0.090891411	0.005852645
L-glutamic acid	1.119840876	0.580704054	0.077332657	0.041604947	0.096773607	0.043300644	0.041828308	0.039074844
2-amino-2-methylpropanoate	1.118187088	0.582261734	0.121796407	0.009818818	0.184034517	0.046975053	0.036095081	0.034617086
3-aminoisobutanoate	1.118187088	0.582261734	0.121796407	0.009818818	0.184034517	0.046975053	0.036095081	0.034617086
D-α-aminobutyric acid	1.118187088	0.582261734	0.121796407	0.009818818	0.184034517	0.046975053	0.036095081	0.034617086
L-(+)-2-Aminobutyric acid	1.118187088	0.582261734	0.121796407	0.009818818	0.184034517	0.046975053	0.036095081	0.034617086
5'-Methylthioadenosine	0.997693195	0.991799608	0.409313149	0.053244526	0.331062712	0.034644512	0.010904041	0.040966945
4-Guanidinobutanoic acid	0.855665922	0.52589644	12.29090872	0.014152117	10.51650961	3.68725E-05	16.49021266	0.082465636
5-amino-1-[3,4-dihydroxy-5-(hydroxymethyl)oxolan-2-yl]imidazole-4-carboxamide	3.639715906	0.00628222	3.449020449	0.302305656	3.084503551	0.036719393	3.2996711	0.246876587
Putrescine	2.141896053	0.143502079	103.5827064	0.010566997	77.11744813	0.002413465	1.23679359	0.414736602
L-Histidine	1.181017928	0.36919145	2.071653071	0.018182106	1.523050139	0.033820853	1.113057314	0.559144325
13(S)-HPOT	0.965853491	0.593941183	0.871471244	0.144028469	0.778826866	0.005050769	0.982235445	0.752401271
N-acetyl Putrescine	1.202678012	0.410804131	2.359640024	0.015472336	1.794095397	0.04152126	0.978722199	0.924515201
3-hydroxy isovaleric acid	1.196164241	0.622202795	4.32403076	0.007884285	2.818673748	0.006928969	5.131755898	ND
L-aspartate	1.104928824	0.63229002	0.144665156	0.048936221	0.151836207	0.05019662	0.103504291	0.045865098
7-methyl Guanine	1.319495289	0.297089595	2.191051885	0.029321125	1.74013682	0.051158818	4.349613076	0.002704836
Galacturonic acid	0.492289209	0.068718096	0.454333956	0.019489525	0.612152626	0.055662939	0.634961998	0.068074484
5-oxo-proline	0.968112431	0.852101206	0.187743125	0.011336635	0.413077759	0.056783322	0.036371076	0.020862129
pyro Glutamic acid (5-oxoproline)	0.968112431	0.852101206	0.187743125	0.011336635	0.413077759	0.056783322	0.036371076	0.020862129
Pyrrolidonecarboxylic acid	0.968112431	0.852101206	0.187743125	0.011336635	0.413077759	0.056783322	0.036371076	0.020862129
Ile-Val	1.124929035	0.616070102	0.064375647	0.052243634	0.125063684	0.057657013	0.038172665	0.049724915
Val-Leu	1.124929035	0.616070102	0.064375647	0.052243634	0.125063684	0.057657013	0.038172665	0.049724915
Val-Ile	1.124929035	0.616070102	0.064375647	0.052243634	0.125063684	0.057657013	0.038172665	0.049724915

Leu-Val	1.124929035	0.616070102	0.064375647	0.052243634	0.125063684	0.057657013	0.038172665	0.049724915
N-acetyl Leucine	1.073523946	0.792969572	2.912855916	0.006819747	1.980938529	0.061791744	3.224652203	0.004891712
Pantothenic Acid	1.092135683	0.652456821	2.19218583	0.007219408	1.520708161	0.062792727	4.666709108	0.002023426
Asn-Pro	1.635587474	0.032948523	1.463452765	0.089499656	1.423812011	0.084665741	1.27045046	0.206202639
Ser-Thr	1.294384109	0.341042619	0.138537339	0.081356679	0.168945953	0.086386273	ND	ND
Thr-Ser	1.294384109	0.341042619	0.138537339	0.081356679	0.168945953	0.086386273	ND	ND
L-Phenylalanine	1.271443187	0.326476466	0.339910841	0.099637569	0.310396416	0.09324993	0.008284228	0.049597612
L-serine	1.119454938	0.589897855	0.417333943	0.097407282	0.419365444	0.096233274	0.044405976	0.039857296
Indole	1.263208929	0.454885282	0.415749248	0.120525155	0.380526591	0.102400373	5.092328311	0.003857233
IS-Trp	1.049760998	0.2838379	0.899342514	0.431529087	0.886349576	0.116515637	0.858581898	0.00805224
L-erythro-4-Hydroxyglutamate	1.284859561	0.321229202	2.022489261	0.019443472	1.507286112	0.129938353	2.359033113	0.012779067
Pterin	1.284859561	0.321229202	2.022489261	0.019443472	1.507286112	0.129938353	2.359033113	0.012779067
3-Indoleacetic Acid	0.81139784	0.448773124	1.85070727	0.027266676	1.425879126	0.138617677	2.788520828	0.005261224
5-hydroxy Indoleacetaldehyde	0.81139784	0.448773124	1.85070727	0.027266676	1.425879126	0.138617677	2.788520828	0.005261224
Indoleacetic acid	0.81139784	0.448773124	1.85070727	0.027266676	1.425879126	0.138617677	2.788520828	0.005261224
Guanosine 3',5'-cylic monophosphate (cGMP)	1.32625179	0.257973379	1.824904617	0.041605837	1.515922347	0.165025751	1.555099701	0.110321201
L-lysine	1.117091197	0.658336797	0.646489944	0.258480008	0.554884567	0.188534968	0.024112387	0.049931873
Trimethylacetic acid	3.248741096	0.054330991	2.651000957	0.111731309	1.638125022	0.201722947	5.096506225	0.014261241
Glyceric acid	0.662582131	0.150833822	0.596183177	0.077224997	0.771027353	0.222234006	0.13634197	0.028318686
Leucinic acid; Hydroxyisocaproic acid	2.780690861	ND	7.505161606	0.01367008	4.048933888	0.275987425	1.574116782	ND
5-hydroxy Lysine	1.313915299	0.360185188	2.31469727	0.00846175	0.674721861	0.279636709	4.268782121	0.001826408
Choline	1.024692741	0.439552928	0.936297624	0.725549381	1.15690996	0.287321348	0.84098389	0.012041801
N1-Acetylspermidine	0.759570665	0.323228377	1.382486845	0.222666885	0.782177181	0.325456716	0.258549082	0.017098793
Imidazoleacetic acid	0.958534094	0.921974021	1.337258532	0.476172031	1.351598915	0.453076574	7.484013808	0.007624278
Histamine	1.326321705	0.279255122	1.88843376	0.028496721	1.203622203	0.457454555	1.753292928	0.052386431

3-(2-hydroxyphenyl)propanoate	0.334802698	0.025626434	2.239651274	0.041713963	1.342945744	0.46138225	0.978822199	0.93546318
Ile-Lys	1.423092701	0.18444866	1.236384529	0.453147867	1.137450397	0.630648449	0.091230667	0.023243107
Eicosanoic acid (20:1)	0.878155833	0.049824681	0.902424549	0.220781084	0.970676015	0.714298359	1.147957417	0.227872526
Uracil	1.23482782	0.685641082	1.057865278	0.850399496	0.892640302	0.75339564	2.966260239	0.036086493
Citrulline	1.100157484	0.583945976	0.898891239	0.658492169	0.950692105	0.823038749	0.370343765	0.013489059
Adenine	1.285645229	0.12714525	4.993667914	0.005182231	0.986845747	0.956447516	8.906877185	0.000279769
Oxypurinol	1.29025128	0.371772788	ND	ND	ND	ND	2.039728865	0.046691207
Xanthine	1.29025128	0.371772788	ND	ND	ND	ND	2.039728865	0.046691207

Note: Ratio >1, increase production as compared to 0 h;

<1, decrease production as compared to 0 h

ND, metabolite not detected

Supplementary Table 6. List of commercially available metabolites used in the growth promotion assay on nine representative *Chloroflexi* and *Bacteroidetes* strains.

Compound	CAS	Concentration 1 (mg/ml)	Concentration 2 (mg/ml)	Concentration 3 (mg/ml)
Cytosine	71-30-7	1	0.1	0.01
Adenine	73-24-5	1	0.1	0.01
Uracil	66-22-8	1	0.1	0.01
2'-Deoxycytidine	207121-53-7	10	1	0.1
3'-Deoxycytidine	7057-33-2	10	1	0.1
Deoxycytidine	1186526-91-9	1	0.1	0.01
Indole	120-72-9	1	0.1	0.01
3-Iindoleacetic acid	87-51-4	1	0.1	0.01
Isopentenyladenine	2365-40-4	1	0.1	0.01
Hydroxypyruvate	1113-60-6	0.1	0.01	0.001
Putrescine	110-60-1	10	0.01	0.001
N-acetyl Glutamic acid	19146-55-5	1	0.1	0.01
Muramic acid	1114-41-6	0.1	0.01	0.001
Pantothenic acid	599-54-2	10	1	0.1
Imidazole acetic acid	70615-26-8	10	1	0.1

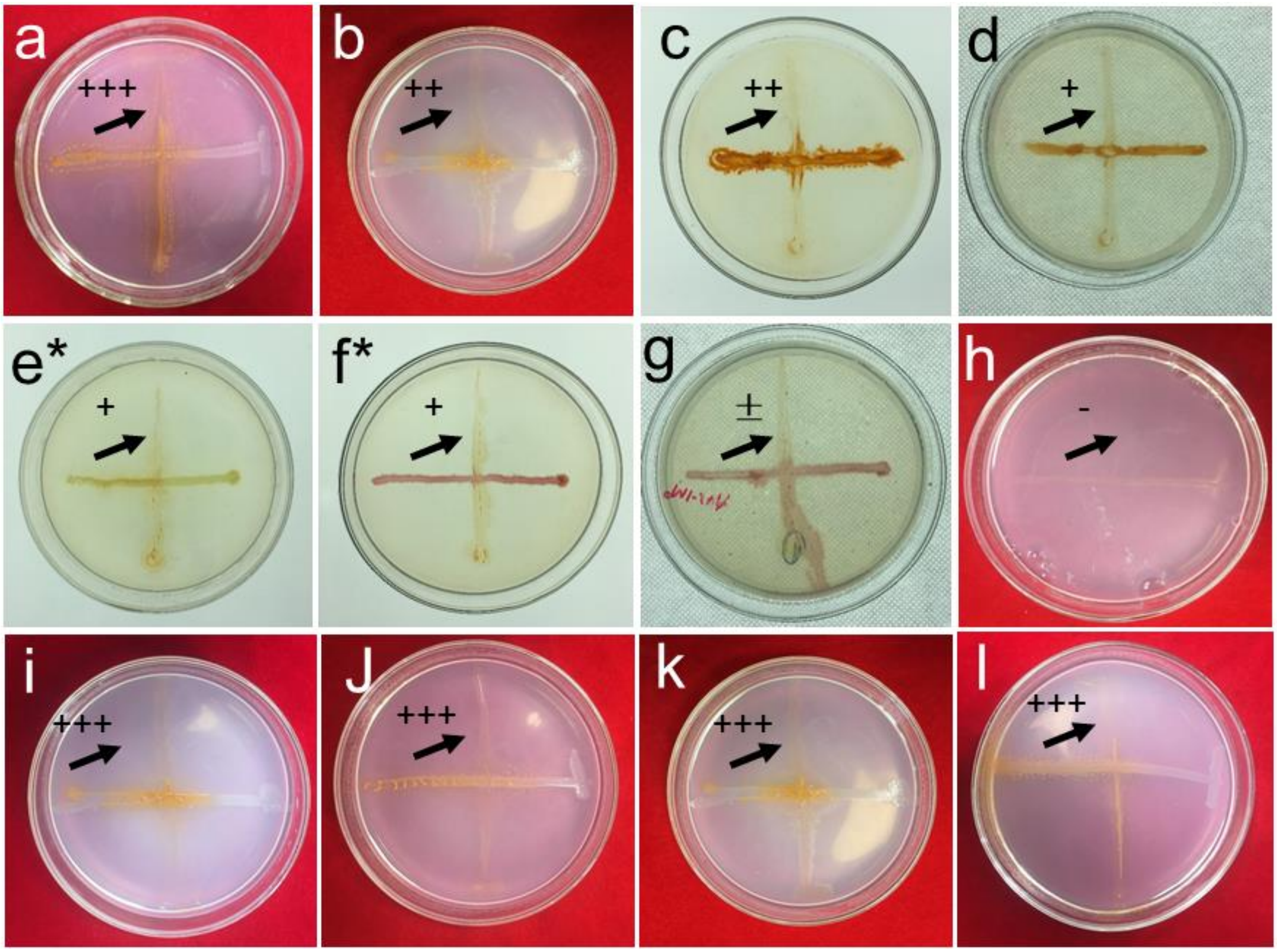

Supplementary Figure 1. Cross-streak analyses for growth promotion using eleven key node bacteria on *Chloroflexus* sp. SYSU G00190R. The putative key nodes bacteria were streaked on the horizontal direction while the indicator *Chloroflexus* sp. SYSU G00190R on the vertical direction. Both strains are incubated at either 45°C or 55°C for 1 week under dark conditions. The enhancement of growth was represented by the (+) sign.

Note: +++, good activity; ++, moderate activity; +, weak activity; -, no growth enhancement

*incubation at 45°C.

Key node strains:

(a) *Tepidimonas* sp. SYSU G00190W (*Tepidimonas ignava* SPS-1037^T, 99.79% 16S rRNA gene sequence identity)

(b) *Tepidimonas sp*. SYSU G00470 (*Tepidimonas taiwanensis* I1-1^T, 98.27%)

(c) Geobacillus sp. SYSU G00408 (*Geobacillus vulcani* 3S-1^T, 99.88%)

(d) Geobacillus sp. SYSU G00405 (*Geobacillus kaustophilus* NBRC 102445^T, 100%)

(e) *Sphingomonas* sp. SYSU G00007^T

(f) Meiothermus sp. SYSU GA4201 (*Meiothermus hypogaeus* DSM 23238^T, 99.35%)

(g) Meiothermus sp. SYSU GA4201 (*Meiothermus hypogaeus* DSM 23238^T, 99.35%)

(h) *Thermus* sp. SYSU G00458. (*Thermus arciformis* CGMCC 1.6992^T, 99.87%)

(i) *Tepidimonas ignava* SPS-1037^T

(j) *Tepidimonas taiwanensis* I1-1^T

(k) *Tepidimonas sediminis* YIM 72259^T

(l) *Tepidimonas alkaliphilus* YIM 72238^T

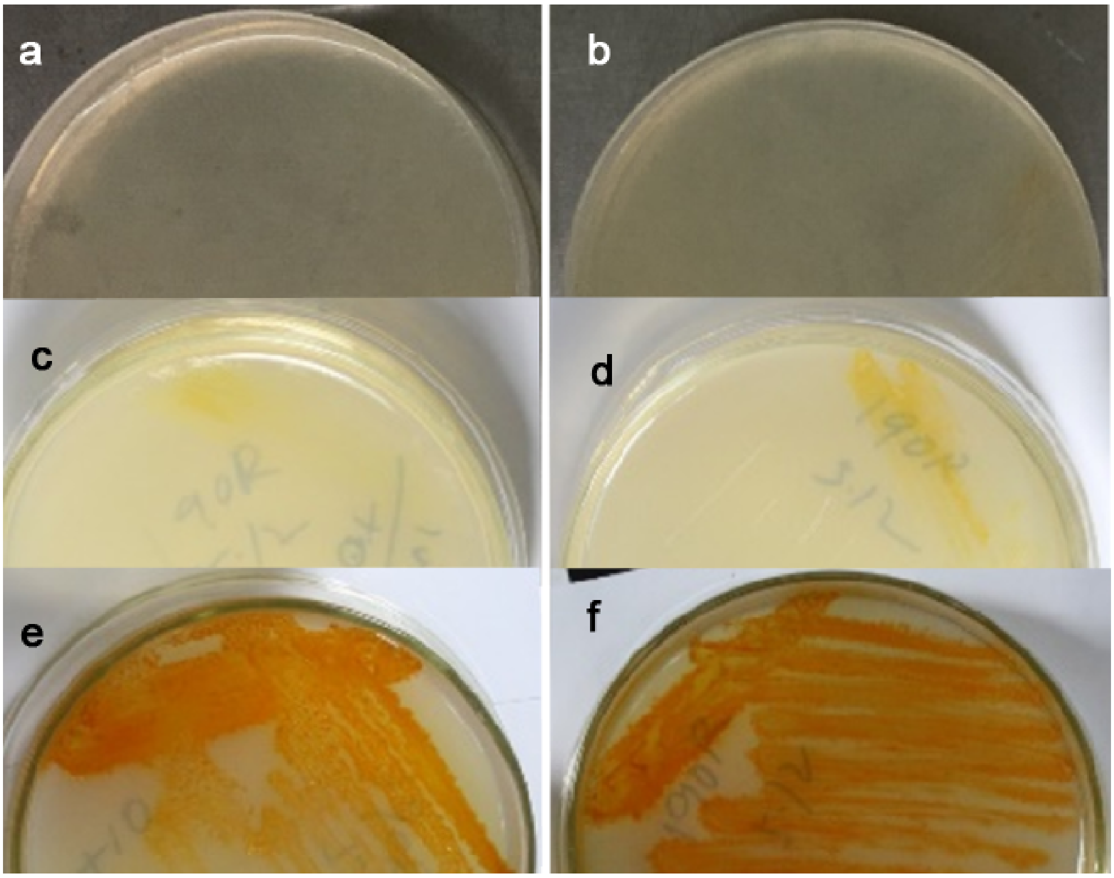

Supplementary Figure 2. Comparison of the growth of the indicator *Chloroflexus* sp. strain G00190R in R2A agar and media supplemented with cell free extract or spent culture supernatants of *Tepidimonas* sp. strain 190W following incubation at 55ºC for 3 days of incubation

Note:
a. R2A agar
b. R2A agar + cell free extract
c. R2A agar + 1% supernatant
d. R2A agar + 5% supernatant
e. R2A agar + 10% supernatant
f. R2A agar + 20% supernatant

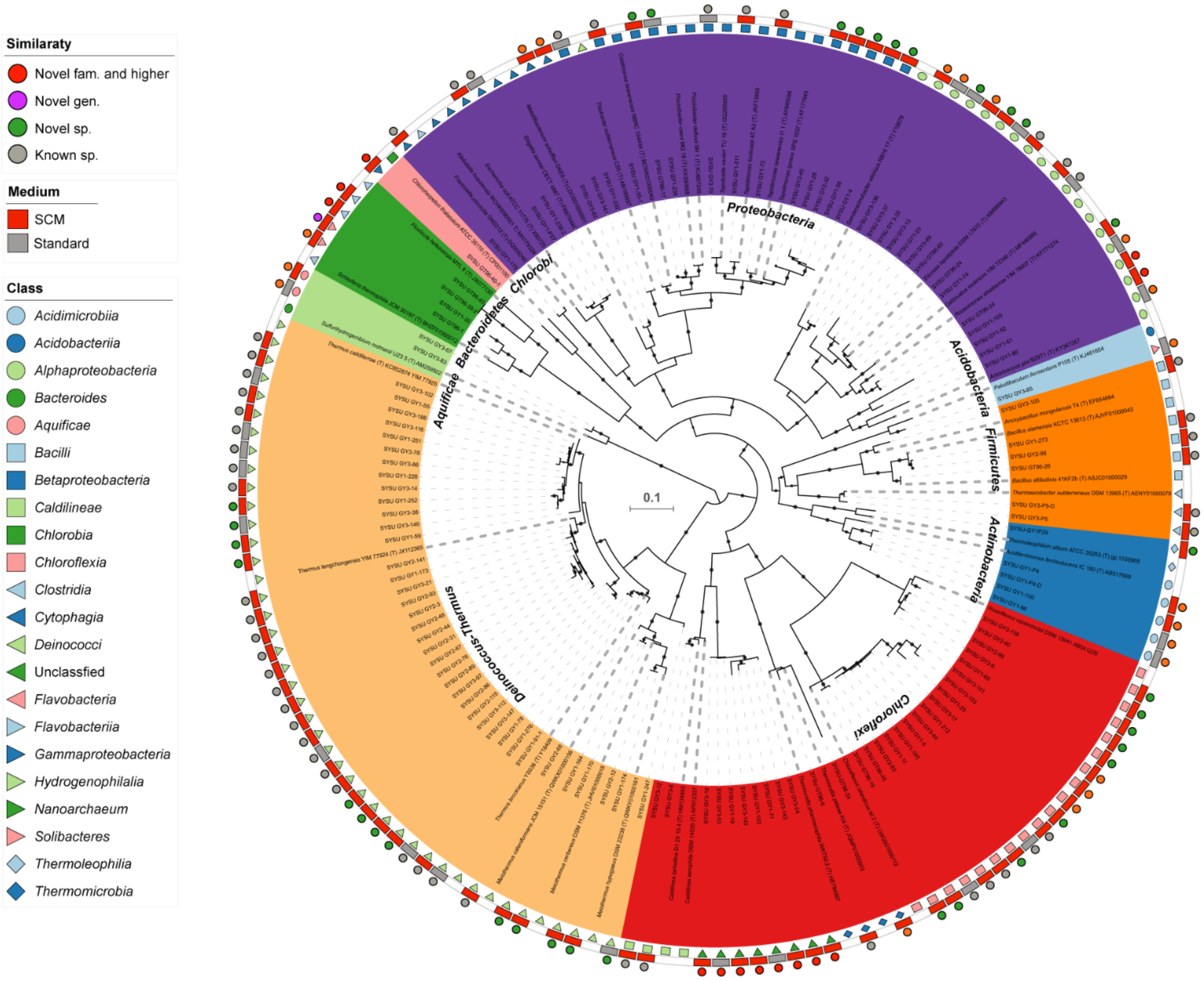

Supplementary Figure 3. Maximum likelihood tree showing the phylogenetic positions of the cultivated isolates determined in this study.

The filled circles in the outermost ring represent the taxonomic ranks of the isolates based on the 16S rRNA sequence identities. Bootstrap values higher than 50 are marked in black circles at the nodes of the phylogenetic tree.

.

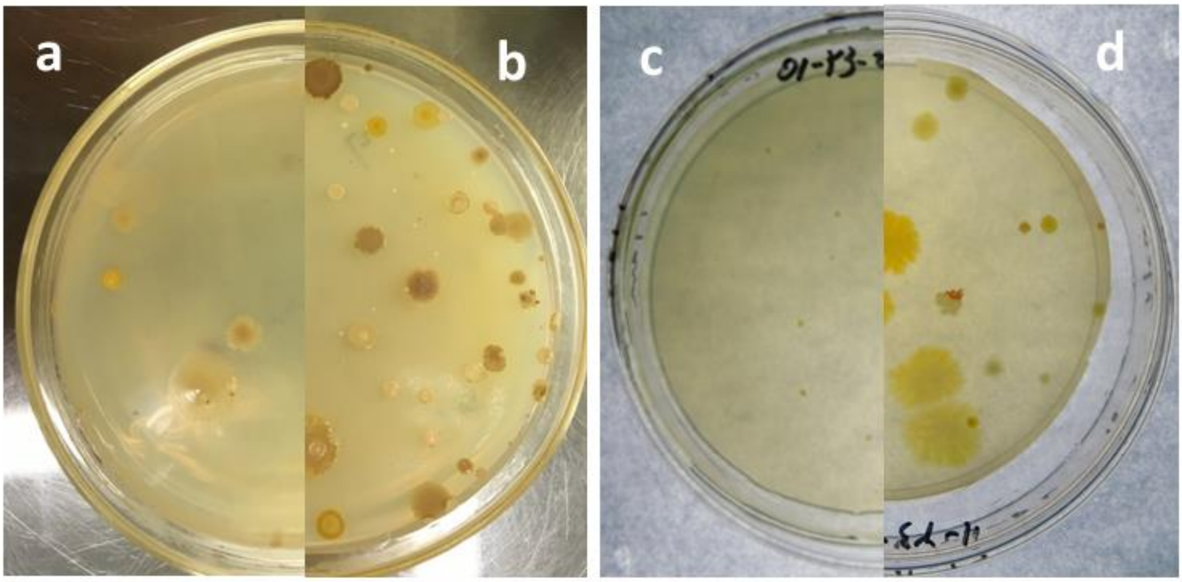

Supplementary Figure 4. Representative isolation plates comparing the difference between traditional (a, c) and SCM-based (b, d) isolation methods. The pictures of the isolation plates shown below are taken after plating the Y3 samples and incubating for 1 week at 55°C

Media used:

(a) R2A agar

(b) R2A agar + 10% spent culture supernatant

(c) *Thermus* 162 agar

(d) *Thermus* 162 agar + 10% spent culture supernatant

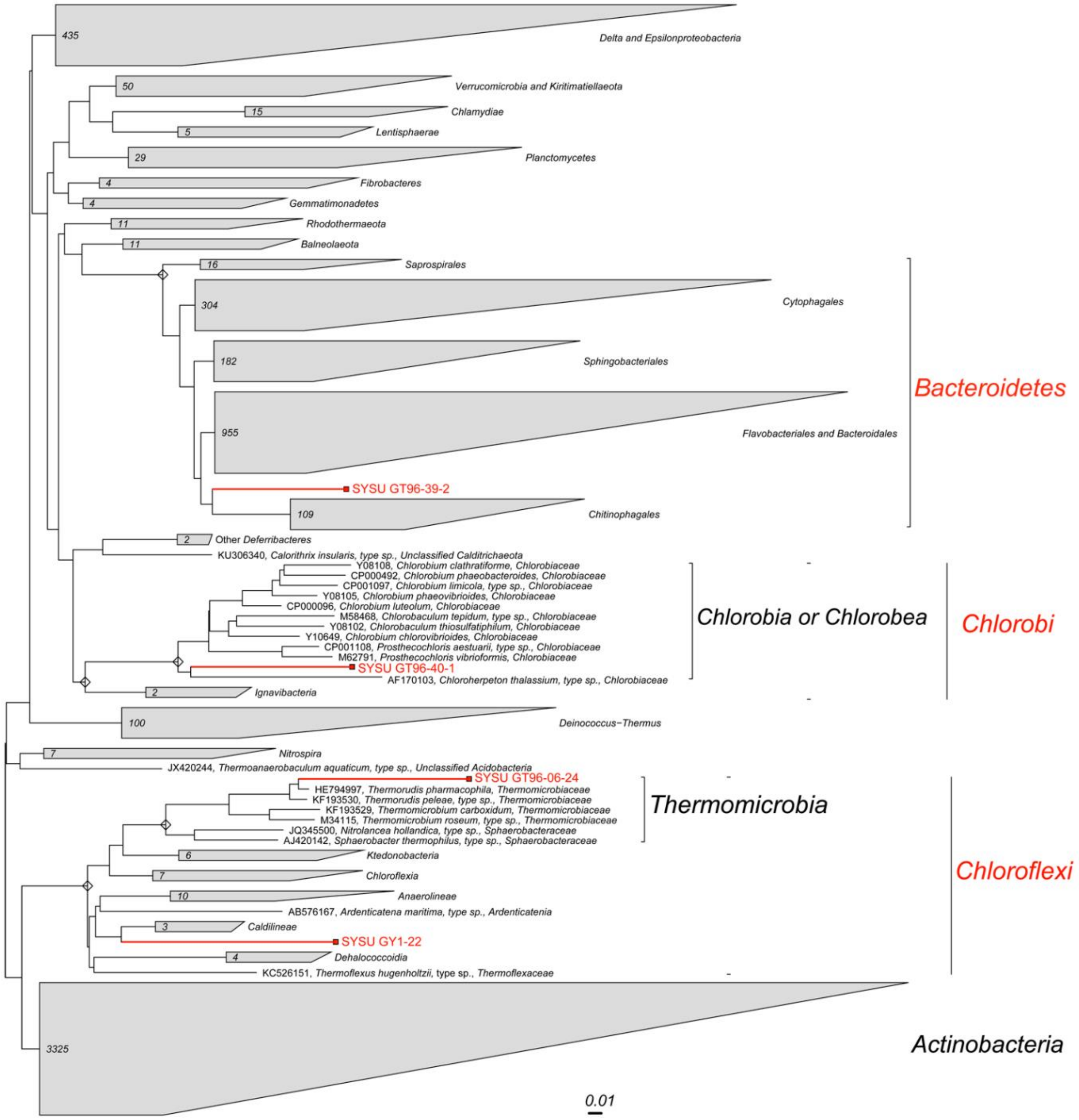

Supplementary Figure 5. Phylogenetic positions of the four strains representing putative novel lineages in the ARB all species tree.

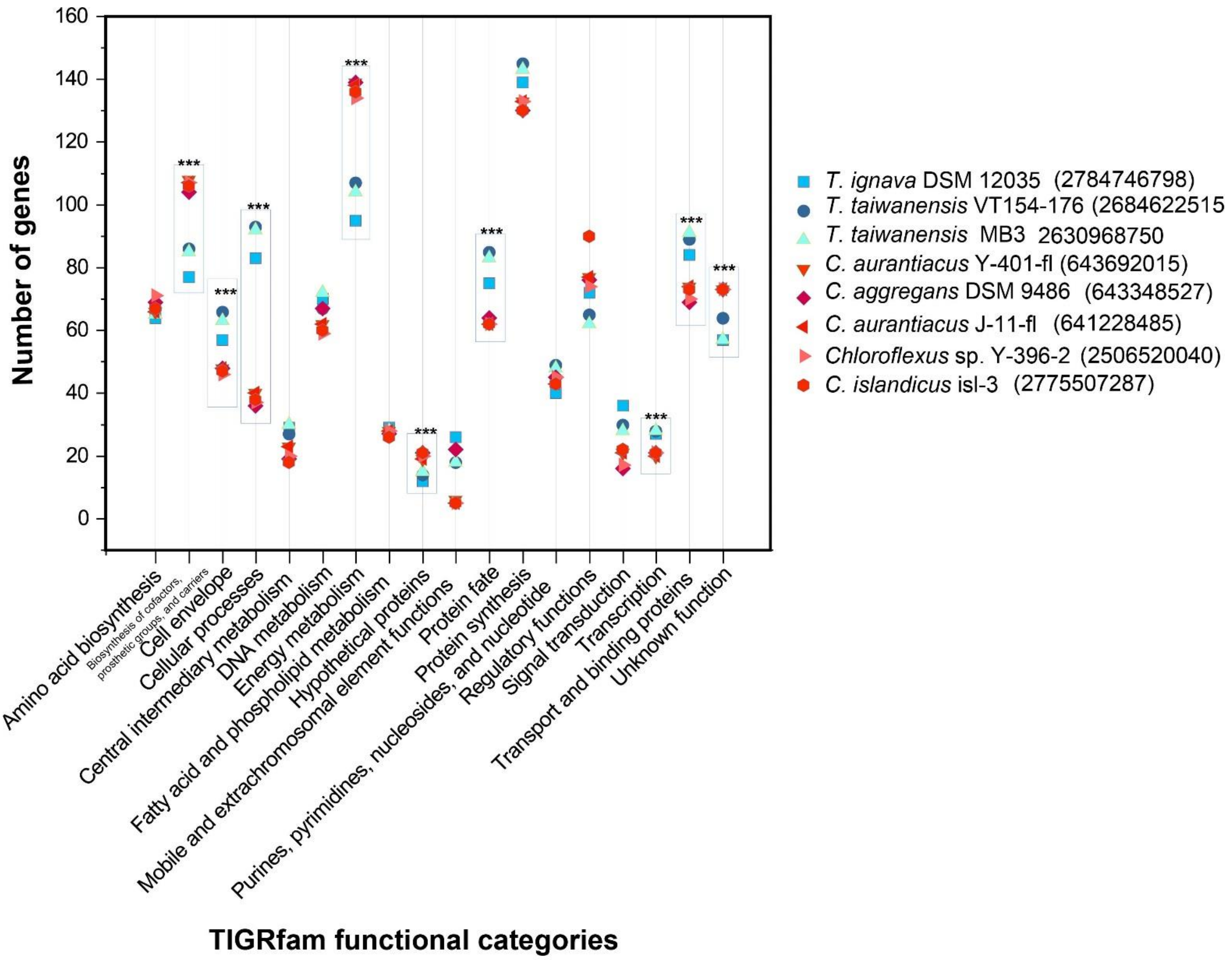

Supplementary Figure 6. Functional annotation of the genomes of *Tepidimonas* and *Chloroflexus* spp.

Categorization of the function of each protein-coding gene was based on the TIGR assignments. *** suggests significant differences between these two microbial groups at $p<0.001$. The colours of these labels show the related species.

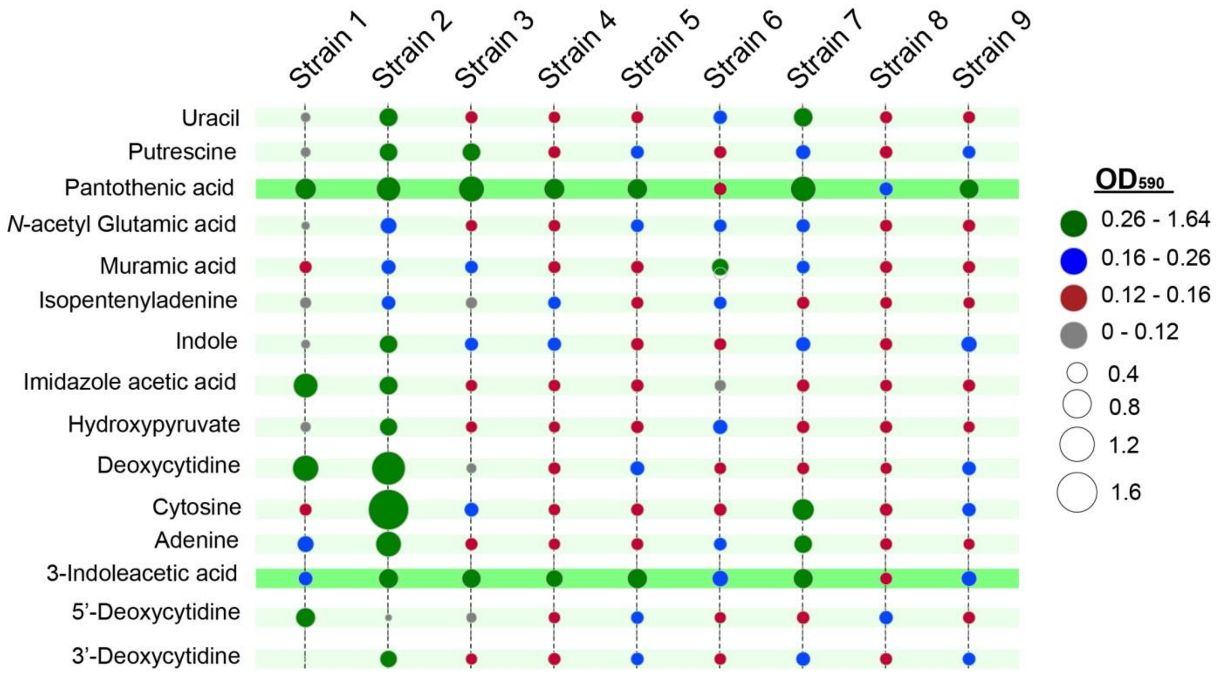

Supplementary Figure 7. Growth promotion assay of metabolites.
Each metabolite was tested in 3 concentration gradients (Table S6) and the most effective concentration as represented by the highest OD for each metabolite is selected for the interpretation of the growth effect. The circle represents the growth OD of each representative strain by the tested metabolite. Color circle depicts range (listed on the figure caption) on which the growth OD falls and the size the general depiction of the actual OD for each strain. The horizontal background line represents the metabolites with common growth promoting effect.

Strain 1: **SYSU GY1-5** [*Chloroflexus islandicus* isl-2^T, 98.67% 16S rRNA gene sequence identity, *Chloroflexi*];

Strain 2: **SYSU GY3-101** [*Chloroflexus islandicus* isl-2^T, 93.39%, *Chloroflexi*];

Strain 3: **SYSU GY2-6** [*Roseiflexus castenholzii* DSM 13941^T, 96.24%, *Chloroflexi*];

Strain 4: **SYSU GY2-90** [*Roseiflexus castenholzii* DSM 13941^T, 96.07%, *Chloroflexi*];

Strain 5: **SYSU GY3-144** [uncultured *Roseiflexus* clone DTM38 (EF205514), 95.69%, *Chloroflexi*];

Strain 6: **SYSU GY1-22** [uncultured bacterium SK47 (AY753393), 99.35%, *Chloroflexi*];

Strain 7: **SYSU GY2-54** [uncultured bacterium SK47 (AY753393), 99.23%, *Chloroflexi*];

Strain 8: **SYSU GT96-06-24** [uncultured *Chloroflexi* clone AE1b_G7, 98.8%, *Chloroflexi*];

Strain 9: **SYSU GT96-39-2** [uncultured bacterium clone NBDTU1 (FJ529921), 89.23%, *Bacteroidetes*].
